# Community-engaged participatory methods for embedding equity in trial design

**DOI:** 10.1186/s13063-026-09820-2

**Published:** 2026-05-29

**Authors:** Martina Svobodova, Nina Jacob, Catherine Lamont Robinson, Becca Thomas, Harley Bryan, Jeremy Segrott, Sarah Bridges, Mashmooma Din, Allan Herbert, Islah Hamad, Aysha H. Ilias, Rena Zhang, Sudipta Bandyopadhyay, Kense Hayan

**Affiliations:** 1 Centre for Trials Research, College of Biomedical & Life Sciences, Cardiff University, Neuadd Meirionnydd, Heath Park, Cardiff, CF14 4YS UK; 2 Centre for Adult Social Care Research, Cardiff University, Cardiff, UK; 3 Social and Community Medicine, Bristol Medical School, University of Bristol, Bristol, UK; 4 Cardiff University, Cardiff, UK; 5 South Riverside Community Development Centre, Cardiff, UK

## Abstract

**Background:**

Certain populations are consistently under-represented in randomised controlled trials (RCTs), including people from minority ethnic backgrounds and those experiencing socioeconomic disadvantage, language barriers, or marginalisation within health systems. This under-representation contributes to widening health inequities. While UK guidance such as the National Institute for Health and Care Research INCLUDE resources supports more inclusive research, practical methodologies for embedding equity in trial design remain limited. This paper draws on two linked public involvement projects: *Talking Trials* (2021–2023), working with ethnically diverse urban communities in Cardiff, and *Let’s Talk Research* (2025), working with residents in a socioeconomically disadvantaged rural Welsh community.

**Methods:**

Both projects used participatory methods including arts-based activities, deliberative dialogue, and co-production. *Talking Trials* incorporated thematic analysis of workshop reflections; both projects generated learning through reflective discussions with community partners.

**Results:**

Community-based public contributors identified features that made research feel relevant and equitable: trusted relationships, time for dialogue, and support to understand complex concepts. Creative and dialogue-based approaches made research concepts accessible, reduced power imbalances, and supported contributors to move from unfamiliarity to active involvement. Sustained structures, such as advisory groups and community connectors, were important for maintaining trust and enabling continued engagement. Context strongly shaped involvement, with different forms of disadvantage requiring different types of support.

**Conclusions:**

Community-engaged, arts-based, and context-sensitive methods can meaningfully enhance equity in trial design. Embedding involvement early, investing in trusted partnerships, and sustaining long-term structures support more relevant, acceptable, and inclusive trials. These findings offer practical methodologies for trial teams seeking to embed equity from the outset.

**Supplementary Information:**

The online version contains supplementary material available at 10.1186/s13063-026-09820-2.

## Background/rationale

Minority ethnic communities and people experiencing socioeconomic disadvantage or structural barriers to healthcare remain under-represented in randomised controlled trials (RCTs). In UK policy, such groups are often described as ‘underserved’, reflecting their relative exclusion from research and health systems despite greater health need. The persistent under-representation of underserved groups in randomised controlled trials (RCTs) is well documented and remains a significant contributor to health inequities [1–3]. Ethnic minority communities were notably under-represented in COVID-19 research despite the greater COVID-19 burden experienced by these populations [4]. Both individual-level socioeconomic disadvantage (e.g. lower income, education, or employment insecurity) and residence in areas of high deprivation have been associated with lower participation in UK clinical trials [5]. Importantly, these structural and interpersonal factors intersect: patients from more deprived backgrounds have been shown to receive shorter and less detailed trial consultations, reinforcing how socioeconomic position shapes opportunities for inclusion in RCTs [6]. These patterns do not operate independently: intersectional combinations of ethnicity, deprivation, migration status, language, and gender shape distinct and overlapping barriers to trial participation [2].

This under-representation applies not only to trial participation but also to the contribution by these communities to research design and delivery [7]. This exclusion contributes to continuing inequalities in the evidence base, as well as in healthcare provision and health outcomes within these populations.

The National Institute for Health and Care Research (NIHR), the UK’s largest funder of health and care research, identified many of these groups as underserved and produced practical resources, such as the INCLUDE Ethnicity Framework and Trial Forge Guidance 3, to support their inclusion in trials [8, 9]. Evaluation of the Framework demonstrated that, while researchers recognise the importance of increasing engagement and recruitment of underserved groups, further resources, institutional commitment, and broader structural change are needed to support effective implementation. The evaluation further recognised involvement of patients, public, and communities in the research process was the most common strategy for improving the participation of underserved groups in health research [10]. However, practical approaches for putting these principles into action in real-world trial design remain limited.

A realist review of research participation decisions among people from diverse ethnic and cultural backgrounds highlighted the need for a more inclusive infrastructure and an increased social presence of researchers within communities [11]. Broader community-led solutions are increasingly recognised as central to tackling health inequalities [12], with evidence showing the importance of community-level factors in shaping health outcomes [13]. Community engagement has also been found to be particularly effective in public health interventions for disadvantaged groups [14].

Against this backdrop, *Talking Trials* was established in 2021 as a collaboration between Cardiff University and the South Riverside Community Development Centre (SRCDC). The project brought together contributors from diverse ethnic backgrounds to explore attitudes to health research using arts-based and deliberative approaches [15]. Arts-based and dialogue-led approaches have been shown to support understanding, reduce power imbalances, and create accessible entry points for people with varied literacy, linguistic, or cultural backgrounds [16]. Through co-production workshops, the group generated a set of recommendations outlining how people from minority ethnic communities can shape research development, delivery, and dissemination [17]. This work highlighted the importance of relational, trust-based approaches to involvement, particularly for communities with historically limited involvement in research.

Building on this foundation, the *Let’s Talk Research* project extended the approach to communities in the South Wales Valleys, working with residents from areas of high socioeconomic deprivation. This enabled exploration of how creative, participatory methodologies can be adapted across different demographic and geographic contexts. Working in this new setting also made visible how different forms of disadvantage shape the kinds of support, pace, and relational work required for inclusive involvement. Together, these projects contribute practical, community-engaged strategies for building trust, addressing intersectional barriers, and embedding equity into trial design and delivery.

Both initiatives centre around culturally grounded, place-based approaches and demonstrate how inclusive public involvement can shape trial processes from the bottom up. They also highlight how methods may need to be adapted across contexts: what works for one underserved group may not work for another, with intersectionality shaping not only barriers but also potential solutions.

In this paper, we present practical methodologies developed with communities to promote inclusive trial design and provide transferable approaches for research teams committed to embedding equity, trust-building, and context-sensitive public involvement within trial design.

## Methods

### Talking Trials

*Talking Trials* was designed using a collaborative community approach, grounded in principles of co-production, equity, and reciprocity. The project was developed in collaboration with the South Riverside Community Development Centre (SRCDC), a trusted third-sector organisation embedded in an ethnically diverse and socioeconomically disadvantaged area of Cardiff. Recruitment was facilitated through SRCDC’s networks and existing community groups, focussing on individuals from diverse ethnic backgrounds who had limited prior involvement in health research. Participation was based on purposive and convenience sampling through SRCDC networks, with an emphasis on involving individuals who had not previously engaged in health research. This approach reflected the project’s aim to work with people already rooted in local community settings, rather than attempting to recruit a demographically representative sample.

The project established a public involvement advisory group of 20 public contributors, who took part in a series of structured workshops between 2021 and 2023. Workshops were co-facilitated by university researchers, community partners, and an artist-collaborator, and were held in accessible, familiar community venues as well as online. Sessions typically lasted around 2 h and combined whole-group discussion with small-group creative activities. The artist-collaborator supported the design and facilitation of these activities, helping to translate complex research concepts into accessible visual/tactual forms.

The methodology combined arts-based activities with deliberative dialogue to explore perceptions of clinical trials. By deliberative dialogue, we refer to structured, facilitated discussion that encourages participants to engage with evidence, reflect on differing perspectives, and collectively consider implications for research practice. This approach enabled discussion of complex trial concepts (e.g. randomisation, informed consent, inclusion/exclusion criteria, importance of inclusivity in research) in ways that supported understanding and participation among individuals facing language, literacy, or digital exclusion barriers. Creative activities included collage, collective drawing, and visual prompts designed to support expression across different language and literacy levels.

The workshops across both *Talking Trials* and *Let’s Talk Research* followed a consistent structure and agenda, adapted to the local context but maintaining the same core sequence. Each session combined introduction of research concepts, facilitated discussion, and creative activities designed to support reflection and dialogue. A typical workshop included:

1. Introduction of a focal topic (e.g. clinical trials, inclusion criteria, participation)
2. Small-group facilitated discussion to explore initial perceptions and experiences
3. Creative activity (e.g. collage, drawing, visual prompts) to support expression and reflection
4. Whole-group deliberative discussion integrating different perspectives
5. Summary and identification of key insights

For example, in a workshop exploring barriers to research participation, contributors were invited to reflect on why certain communities are systematically excluded from research, drawing on their own community contexts and experiences. The group discussed these issues, followed by a creative activity in which participants used collage materials to represent perceived barriers. These visual outputs were then used as prompts for whole-group discussion, with individuals invited to share and describe their artwork and what it represented to them. This process enabled contributors to reflect on structural and experiential barriers and to identify ways trials could be made more inclusive.

Across sessions, there was a progression from building familiarity with research concepts to more in-depth discussion of inclusivity in trial design and participation. This iterative structure supported contributors to move from initial uncertainty to more confident and active engagement.

A distinctive aspect of the project was the formation of ‘community connectors’, a smaller group of contributors who took on expanded roles in peer-to-peer engagement. Community connectors were identified through ongoing workshop participation and expressed interest in taking on a more active role. Drawing on their own experiences in the *Talking Trials* workshops, these connectors designed and delivered their own sessions within existing community networks, including an ESOL English class, a women’s chat group, a youth group, and a digital-literacy class. Using creative materials as prompts for discussion, they facilitated meaningful dialogue in spaces where contributors would rarely encounter academic research. This ‘in-reach/out-reach’ approach not only extended participation but also reinforced the role of community members as co-creators of knowledge.

The process generated a set of co-produced recommendations on inclusive research practice, which were later formalised into a toolkit for trialists, providing practical guidance on inclusive communication, recruitment, and governance. The *Talking Trials* project also led to the establishment of a standing Research Advisory Group within the Centre for Trials Research. This group comprises public contributors from the project and continues to provide input into trial design, governance, and funding applications beyond the life of the original workshops.

Ethical approval for the 2023 phase of *Talking Trials* was granted by the Cardiff University School of Medicine Research Ethics Committee (SREC reference: 22/81), with informed agreement obtained from all contributors. The 2021 phase of *Talking Trials* and the *Let’s Talk Research* project were undertaken as public involvement and did not require ethical approval, in line with NIHR guidance. Direct quotations in the findings are drawn exclusively from the ethically approved phase; insights *from Let’s Talk Research* draw on reflective notes and collaborative sense-making.

### Let’s Talk Research

The *Let’s Talk Research* project extended this participatory approach into a rural and socioeconomically disadvantaged community in a small ex-mining village in the South Wales Valleys, in partnership with the Caerphilly Parent Network and the local community centre. Recruitment was facilitated through an established parenting group, ensuring activities were grounded in a trusted, pre-existing setting. Approximately 8–10 contributors attended each workshop. Most contributors attended all three workshops, although attendance varied slightly between sessions.

Across three workshops held in 2025, contributors engaged in co-production sessions co-facilitated by researchers and a community artist. These followed the same workshop structure and sequence described above, adapted to the local context. Workshops combined creative art exercises (e.g. collaborative artwork reflecting local identity) and structured discussions to explore community perceptions of health research, and barriers to participation. The community artist co-designed creative exercises with contributors and facilitated visual activities to support expression and reflection.

In the sessions, we focussed on dialogue, prioritising trust-building and reciprocal exchange rather than data collection. Activities took place in a familiar and accessible community centre. Fieldnotes, visual artefacts, and collective reflections were captured, but the primary focus was on building trust, learning from and understanding lived experience, and exploring how creative participatory approaches function in a different demographic and geographic context.

Learning from the public involvement activities across both projects was developed through reflective discussions and collaborative sense-making with community partners, alongside thematic analysis of the Talking Trials workshop data [18]. Together, these processes informed the synthesis presented here.

### Researcher positionality and reflexivity

The research team included academic researchers, community partners, an artist educator (Talking Trials), a place-based artist (Let’s Talk Research), and a student researcher, a composition that was itself a reflexive choice reflecting our commitment to broadening who participates in producing research.

As academics, we occupied positions of institutional authority and were conscious this could shape what contributors felt able to say. Co-facilitation with community partners and creative methods helped to mitigate this, and importantly we participated in the creative activities alongside contributors rather than observing from the outside. One team member (MS) served as Chair of Trustees for the South Riverside Community Development Centre, which supported trust-building but required ongoing reflexive awareness to manage potential role-related influences.

The artist educator (CLR) brought extensive experience of participatory arts practice across health and community settings, including work with patient groups, people with complex learning needs, and communities affected by chronic illness. For *Let’s Talk Research*, the place-based artist (BT) was from a similar post-industrial community, enabling a depth of connection that was a deliberate methodological choice. Throughout both projects, we were mindful of the risk of extractive practice, maintaining dialogue with community partners to ensure the work felt genuinely reciprocal.

## Findings/observations

Across both *Talking Trials* and *Let’s Talk Research*, contributors shared similar reflections about what made research feel accessible, relevant, and worth engaging with. These observations illustrate how involvement grows through trust, shared understanding, and creative dialogue, but also how different community contexts require different forms of support.

**Fig. 1:**
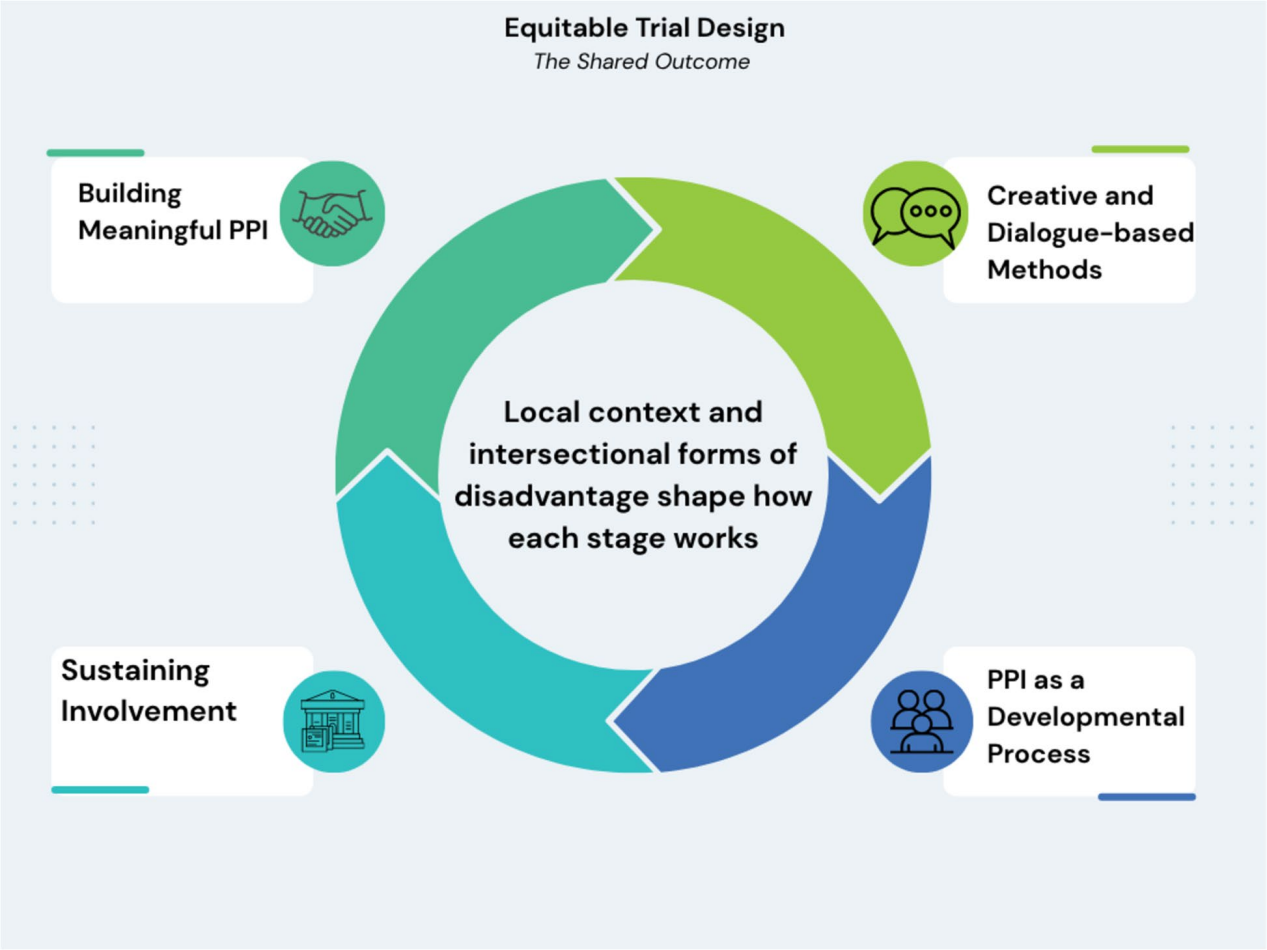
Thematic map illustrating relationships between key themes supporting equitable trial design. Local context and intersectional forms of disadvantage shape how relational, methodological, and temporal processes contribute to equitable trial design

Figure 1 presents a thematic overview of the key themes identified across both projects and their relationships.

The findings presented below combine illustrative quotations from *Talking Trials* with cross-project observations developed through reflective discussion and collaborative sense-making. Where quotations are used, these represent direct participant perspectives; other statements reflect interpretive synthesis across both projects.

### Building meaningful PPI

Across both projects, contributors reflected on what made research feel relevant and worth engaging with, though the quotations below are drawn from *Talking Trials* where formal data collection took place. Meaningful involvement, they suggested, is more than simply inviting participants from underserved groups to participate in research. Inclusion depended on whether contributors felt research was for them and about them. This sense of relevance developed gradually through time, dialogue, and supported opportunities to learn. Many contributors initially perceived research as distant, technical, or irrelevant. As one contributor reflected, ‘It’s more like a confirmation… I knew our community is not involved… But you have to be at the table. Which is completely essential’ (Contributor 1).

In both projects, involvement grew through structured, iterative engagement where contributors often moved from ‘I don’t know anything about that’ to ‘I didn’t realise I could have an opinion on this’. Contributors consistently noted that passive information, such as leaflets, was insufficient. Dialogue and relational exchange were considered essential. One person said, ‘If you just leave a paper, they are passive… They need to be actively involved. Which is why this has worked so well’ (Contributor 2). Another highlighted personal relevance as a key motivator: ‘So my first thing was to increase my knowledge… what is the benefit for me and my family and my community’ (Contributor 3).

When contributors were supported to understand research, they moved from passive recipients to co-facilitators of learning. In *Talking Trials*, community connectors ran sessions for new groups and translated complex concepts creatively through art. One contributor described this progression: ‘I feel that this process, our contribution has been really valued… the workshops make them more informed and they can spread that awareness… when the parents know then the children will know’ (Contributor 4).

These findings highlight that diversifying PPI is a relational and developmental process. Early engagement, shared dialogue, and active capacity-building are essential components that create the conditions for meaningful participation and support trial teams to design studies that feel relevant and accessible from the outset.

### Using creative methods

Creative and dialogue-based approaches helped make research accessible and relatable. Arts-based activities provided a ‘safe space’ for discussing challenging topics. One contributor explained: ‘The artistic creative way is a safe way for us to talk about something that is hard and difficult… It levels us’ (Contributor 1).

These approaches, observed across both projects, enabled contributors to express ideas in ways that did not rely solely on verbal or written explanation and to:

- Communicate more openly with researchers
- Co-explore barriers and motivations for participation
- Engage with research in ways that matched cultural, linguistic, and learning needs

This was particularly evident in *Talking Trials*, where thematic analysis supported these observations. One contributor described the problem-solving benefits of creative engagement: ‘The art lets your ideas come through. It allows you to think more about the ideas you have received and helps you to think about problem solving… to find a solution’ (Contributor 2).

By using creative methods, trial teams can shift PPI from transactional consultation to co-constructed involvement, supporting more equal participation, reducing reliance on technical language, and giving contributors greater agency and confidence in shaping trials.

### Sustaining PPI

A major learning from *Talking Trials* was that one-off engagement, however successful, is fragile. Relationships built during individual projects can quickly dissipate without structures that preserve continuity and trust. Establishing a standing community advisory group created continuity and credibility, allowing contributors to influence trial design, review materials, and act as trusted intermediaries in their communities. This group has since become part of ongoing public involvement infrastructure within the Centre for Trials Research. One contributor highlighted institutional support: ‘It’s got to be about reciprocity… the organization is here and we are doing our job… but none of those could have achieved it without the other… You’ve got a place where people feel comfortable being you’ (Contributor 5).

To sustain meaningful involvement, trial teams need long-term mechanisms rather than project-specific activity. These include standing advisory boards, community connectors, and partnerships that continue across studies. Such structures help maintain trust, support shared learning over time, and strengthen the relevance and accessibility of trial materials and processes. Without these mechanisms, engagement must be rebuilt from the beginning with every new study, and the benefits of earlier involvement are easily lost.

### PPI as a growing process

In *Talking Trials*, and reflected in observations from *Let’s Talk Research*, involvement operated as a developmental process with contributors moving from disconnection and doubt to confidence and curiosity. One contributor observed, ‘They started saying, “We don’t know anything about this,” and now we’re the ones explaining it to others’ (Contributor 1).

This shows that perceived disengagement often reflects a lack of opportunity rather than disinterest. With time and repeated interaction, contributors developed a stronger sense of confidence to contribute and a clearer understanding of how research works. Trial teams that prioritise relationship-building, dialogue, and capacity-building help participants gain understanding, agency, and readiness for future involvement. This shifts the narrative from ‘hard to reach’ to ‘insufficiently engaged’, reframing responsibility towards research systems rather than communities.

PPI becomes cumulative and iterative, with confidence building as contributors see their insights used and valued. Over time, this strengthens the quality and relevance of trial design, as communities become more experienced and confident in contributing to research.

### Context shapes involvement

Across the two projects, it became clear that involvement does not look the same in every community, even when the underlying approach is similar. Context shaped how people understood research, what kinds of support they needed, and what made participation feel safe and worthwhile.

In *Talking Trials*, many conversations centred around experiences of migration, language barriers, and feeling distant from UK health systems. Creative and dialogue-based activities supported confidence-building, but contributors also emphasised the importance of being approached through trusted community settings and of having time to build relationships before feeling able to contribute. Contributors drew on shared cultural experiences to make research concepts relevant for others, and this peer-led approach was a key mechanism for engagement.

In *Let’s Talk Research*, different forms of disadvantage shaped involvement. Here, contributors described long-standing disconnection from health services, limited expectations of being listened to, and a sense that research ‘wasn’t for people like us’. Conversations focussed less on language or cultural adaptation and more on the emotional and practical impact of cumulative socioeconomic disadvantage. Trust-building required a slower pace, more informal dialogue, and reassurance that the work genuinely valued their perspectives and was not just taking information from them. Activities that centred local identity, shared experience, and everyday concerns helped create conditions where people felt comfortable participating.

Taken together, these observations show that there is no single route to inclusive involvement. The same participatory methodology functioned differently across settings because the barriers, expectations, and starting points varied. Approaches that are effective in one community may require adaptation in another. For trial teams, this underscores the importance of attending to place, history, and local relationships when designing involvement activity, and of avoiding assumptions that one model of PPI will work universally.

To support transferability, we synthesised learning from both projects into a set of cross-cutting themes that highlight the approaches used and their implications for inclusive trial design (Table 1). These are not intended as prescriptive steps but as evidence-informed considerations for trial teams working to embed equity from the outset.

**Table 1:** Cross-cutting themes and practical implications for embedding equity in trial design

Theme	Methods/approach	Key insights for trial teams
Building meaningful PPI Relational inclusion	Structured workshops; ongoing dialogue; partnership with trusted community organisations	Trust and relevance develop gradually through repeated interaction—time and appropriate resourcing are essential Early relational work strengthens contributors’ confidence and the quality of their input Passive information alone is insufficient; trials benefit from interactive, conversational engagement
Using creative methods Creative and dialogue-led engagement	Arts-based activities (e.g. collage, drawing); visual prompts; small-group discussion	Creative and visual methods reduce power imbalances and support understanding across diverse literacy and language needs These approaches surface perspectives that may not emerge through verbal discussion alone Trial teams working with communities facing linguistic, cultural, or confidence barriers may find creative methods particularly valuable
Sustaining PPI Long-term structures	Community connectors; standing advisory groups; long-term partnerships	One-off engagement is fragile; continuity matters Ongoing structures enable cumulative learning and maintain trust across studies Sustained involvement enhances the relevance and acceptability of trial materials and processes
PPI as a growing process Developmental involvement	Capacity-building conversations; iterative workshops; peer-to-peer learning	Contributors often move from uncertainty to active participation over time Perceived disengagement often reflects lack of opportunity rather than lack of interest Allowing time for relationship-building increases the depth and quality of involvement
Context shapes involvement Place-sensitive adaptation	Adaptations across urban ethnically diverse and rural socioeconomically disadvantaged contexts	Barriers differ across settings (e.g. migration and language vs. long-term socioeconomic precarity) The same methods function differently in different communities Tailoring involvement to place, history, and local expectations is essential—no single model works everywhere

These are not intended as prescriptive steps but as evidence-informed considerations for trial teams working to embed equity from the outset

## Discussion

Our experience across *Talking Trials* and *Let’s Talk Research* highlights that meaningful inclusion is relational, developmental, and context-specific. Inclusive engagement emerged from sustained communication, capacity-building, and co-production rather than from one-off consultation or project-specific activity. Embedding involvement early in the design phase supported contributors to understand the research process, develop confidence, and offer grounded insights, strengthening the relevance and accessibility of later trial materials.

The *Talking Trials* project illustrates how early, structured involvement combined with creative facilitation can evolve into enduring infrastructure. Initially workshop contributors shifted to advisors, supporting the development of a standing Research Advisory Group at the Centre for Trials Research. This group now aligns with UK Standards for Public Involvement and provides input across multiple trial areas—including methodology, women’s health, infections, and population health. It has also collaborated with other UK PPI groups to inform trial design and funding applications, representing a key mechanism for supporting equitable trial design [19]. These outcomes show that investing in trust, reciprocity, and accessibility can translate initial dialogue into embedded, system-level roles within research governance. They also demonstrate how arts-based and dialogue-led approaches can support contributors to move into advisory roles with increasing confidence and skill.

*Let’s Talk Research* tested these methods in a different context: a socioeconomically disadvantaged rural community. Here, inclusion required adaptation. Barriers were less about language or cultural difference and more about disconnection, low expectations, and cumulative socioeconomic disadvantage. Creative, dialogue-driven approaches were essential for building trust and validating lived experience, but methods that worked well in an urban, ethnically diverse context could not simply be transferred without adjustment. This underscores the importance of intersectional sensitivity: ethnicity, deprivation, and other axes of disadvantage intersect to shape both barriers to participation and the strategies needed to overcome them. Methodological flexibility therefore becomes essential.

Together, the two projects demonstrate that inclusive trial design is not achieved by procedural fixes alone. Time-intensive relational work, arts-based facilitation, and context-specific adaptation were central to enabling meaningful participation. While structured tools—such as equality impact assessments or careful selection of PPI contributors—can strengthen trial proposals, they are most effective when combined with community-led dialogue. For trial teams, this means planning for involvement early, resourcing it appropriately, and recognising that community expertise develops over time. This approach shifts inclusion from an optional enhancement to an integral component of trial methodology, contributing to more equitable trial design and more credible evidence for diverse populations.

## Conclusion

Equitable trials begin at the design stage, long before recruitment, and require deliberate investment in relationships, trust, and context-sensitive methods. Community-led, participatory approaches—particularly arts-based and deliberative techniques—enhance both the ethical and methodological quality of trials, by surfacing perspectives that might otherwise be overlooked.

The *Talking Trials* group demonstrates how co-produced recommendations can evolve into sustained advisory structures that contribute to research governance and fundable outputs, while *Let’s Talk Research* shows the need to adapt approaches to local social and emotional contexts. Both projects illustrate that inclusive trial design is possible, necessary, and beneficial, but it demands early, sustained, and flexible engagement.

For funders, trial teams, and institutions, the implications are clear: inclusion should be resourced, embedded, and valued as a core methodological practice. Investing in community-led, participatory PPI from the outset enhances the relevance, accessibility, and acceptability of trials, while building capacity, trust, and sustainable networks that can shape health research infrastructure across the UK, as exemplified by ongoing collaborations with other PPI groups [19]. This direction of travel aligns with recent NIHR guidance on partnership working with patients and the public, which emphasises long-term relationships, community-led approaches, and structural support for equitable involvement [20].

## Limitations

This paper has several limitations. First, although the manuscript draws on learning from both *Talking Trials* and *Let’s Talk Research*, the qualitative data presented through direct quotations are drawn from *Talking Trials*. This reflects the fact that *Talking Trials* included a more formal, ethically approved programme of workshops with a thematic analysis, whereas *Let’s Talk Research* was undertaken as public involvement and was not designed or consented for traditional qualitative analysis. As a result, insights from the Valleys context rely on reflective notes and collective sense-making rather than verbatim participant data.

Second, both projects involved relatively small contributor groups situated within specific community contexts. The intention was not to generate generalisable findings, but rather to explore how community-led and creative approaches function in real settings. Nevertheless, this means that the methodological insights presented here may require further testing or adaptation in other communities.

Third, the work was embedded within established community partnerships, which may not be directly replicable in places where relationships between researchers and communities are less developed. The relational and trust-based components that underpinned involvement in both projects require time and institutional support, and may be more challenging to deliver within short funding cycles.

Despite these limitations, the synthesis presented here offers practical guidance on how community-led, creative, and context-sensitive approaches can strengthen trial design and contribute to more equitable involvement across diverse settings.

## Supplementary Information

Supplementary Material 1.

## Data Availability

Data supporting the findings of this study consist primarily of public involvement reflections and workshop materials that were not collected as research data. These materials are therefore not publicly shareable. Summary materials or illustrative examples may be available from the authors on reasonable request **.**
