# Supplementary material for: Community-engaged participatory methods for embedding equity in trial design
Source: Trials. 2026 May 29;27:512. doi: 10.1186/s13063-026-09820-2 (PMC13411309; doi:10.1186/s13063-026-09820-2)

Talking Trials Workshops: Images of the creative workshop activities

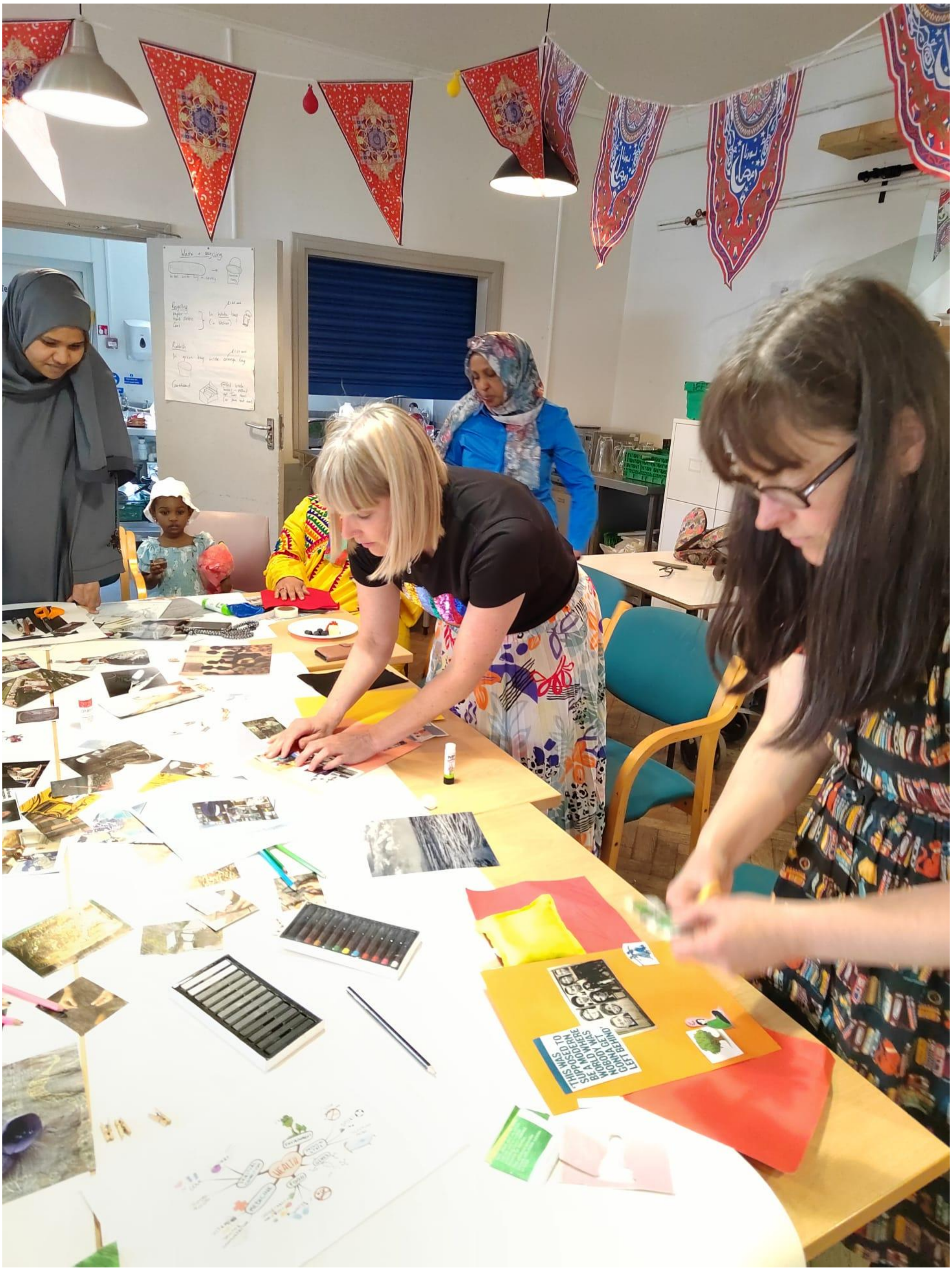

Talking Trials Toolkit
final 5th September 2

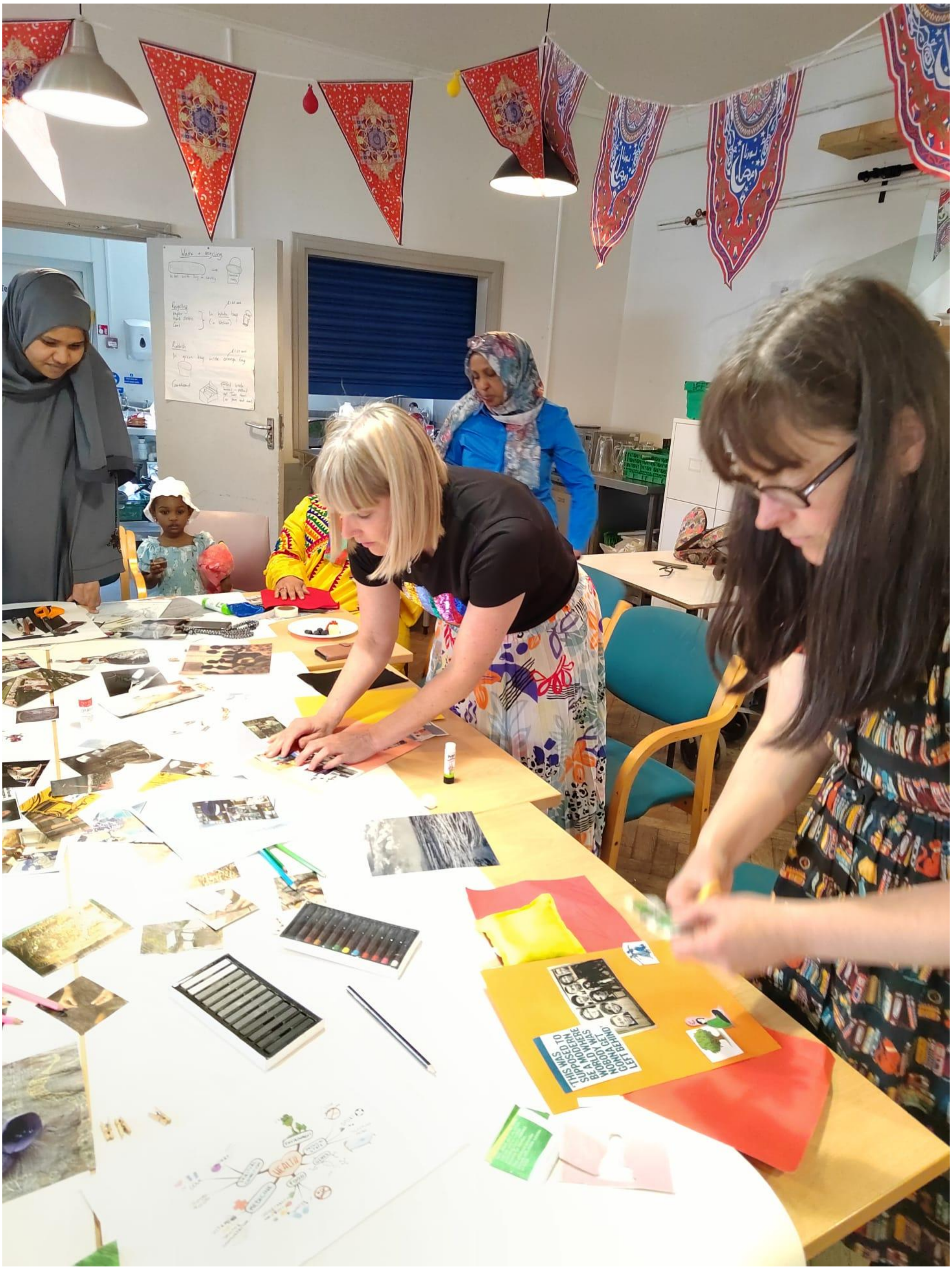

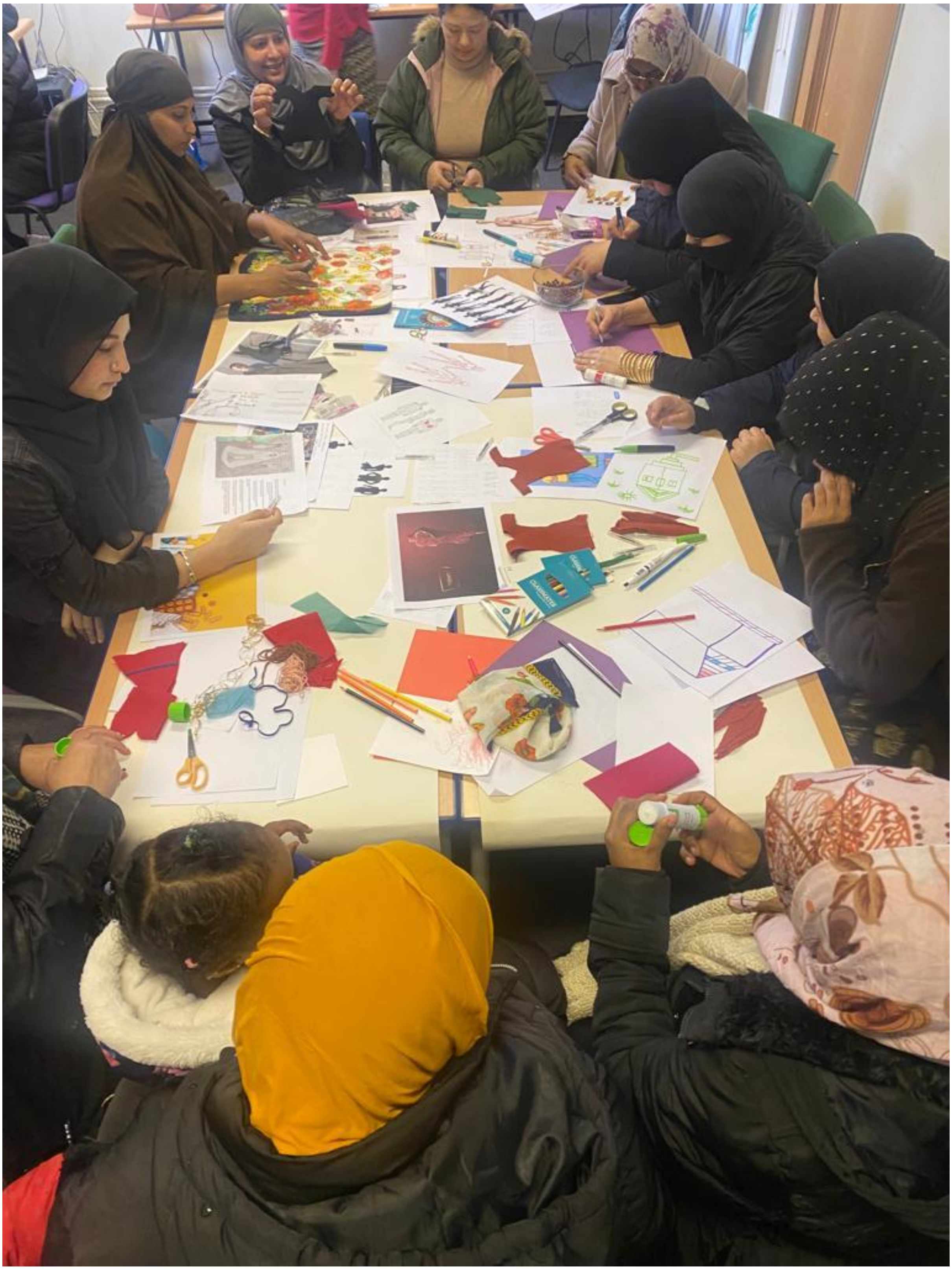

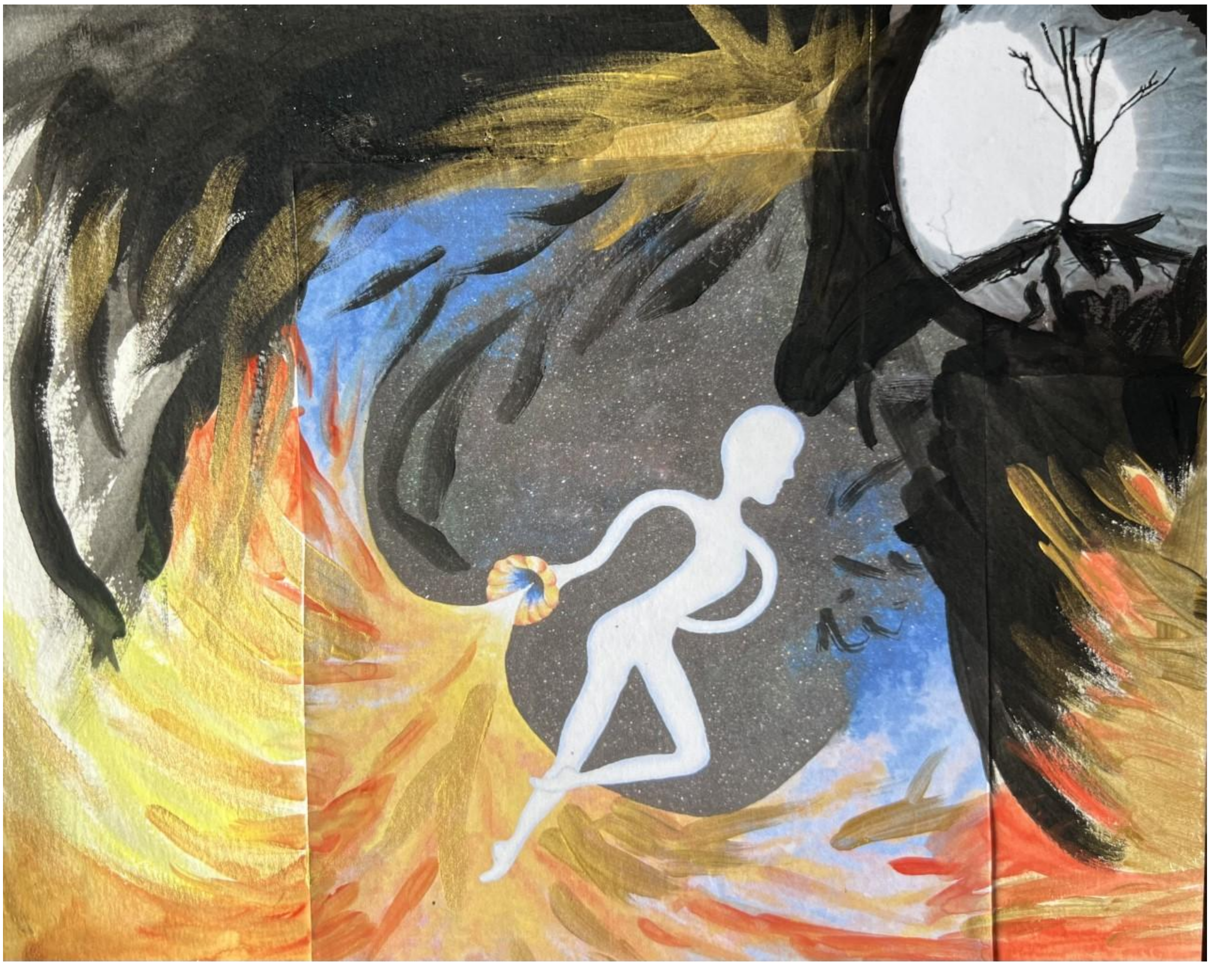

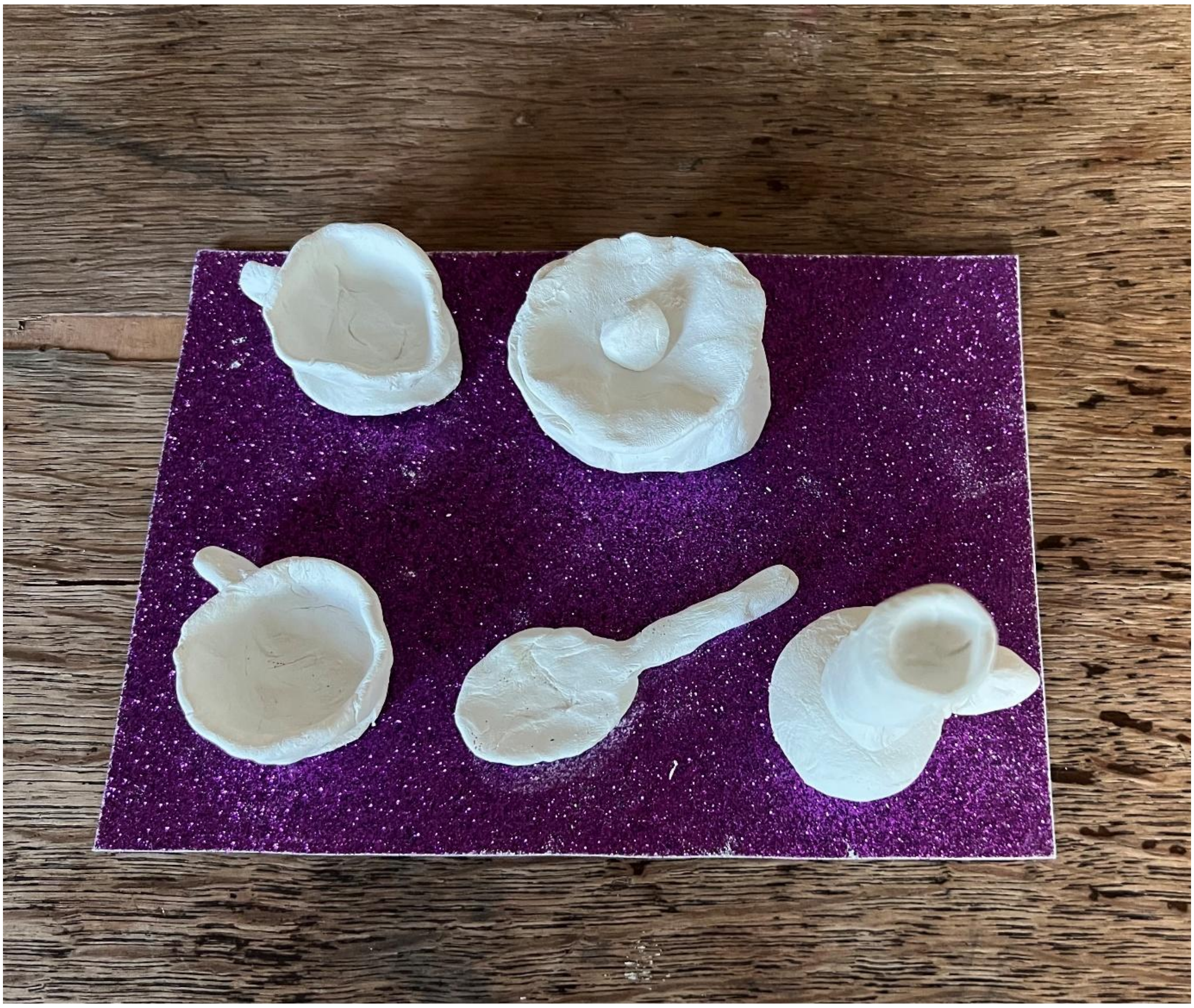

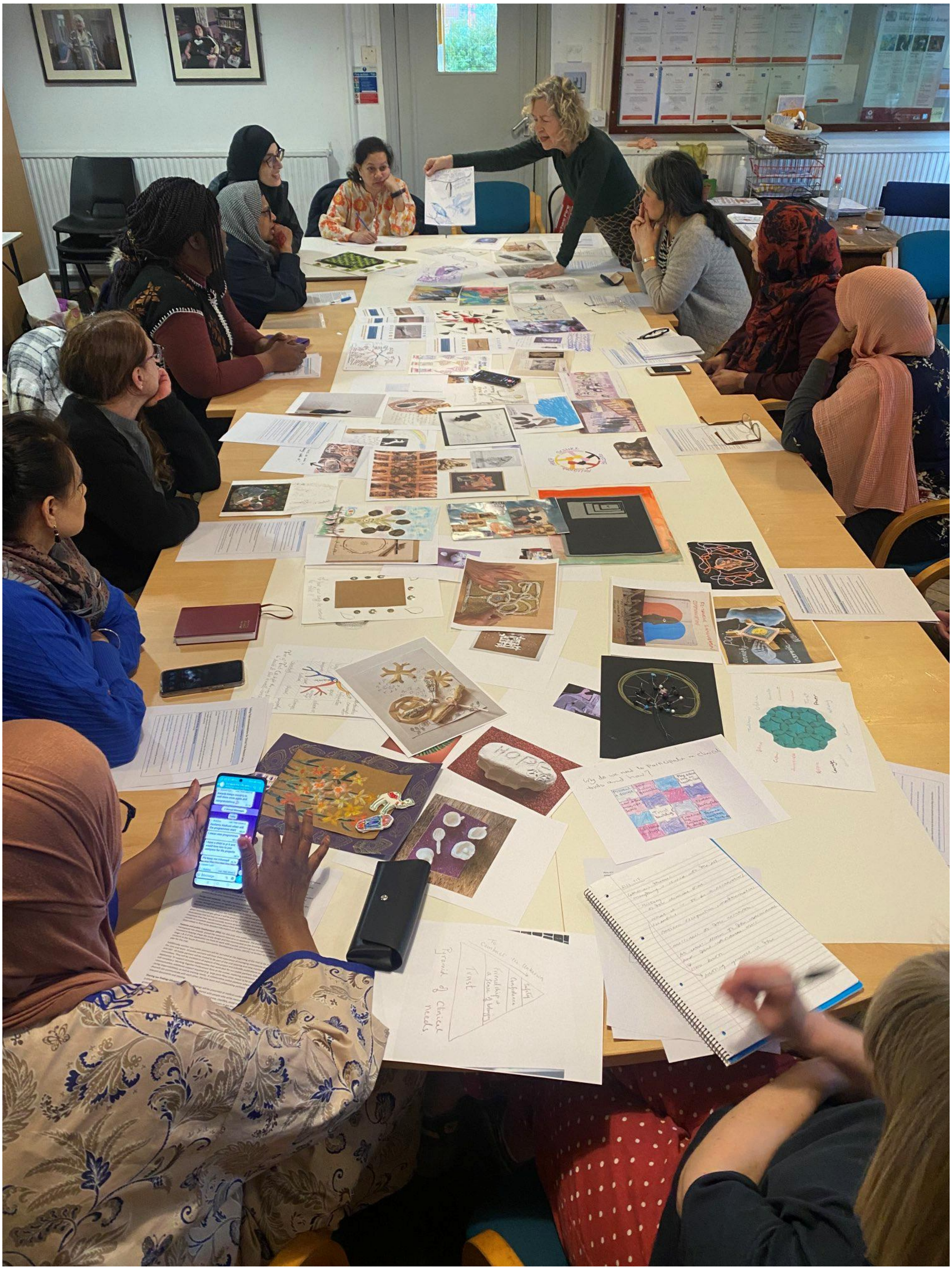

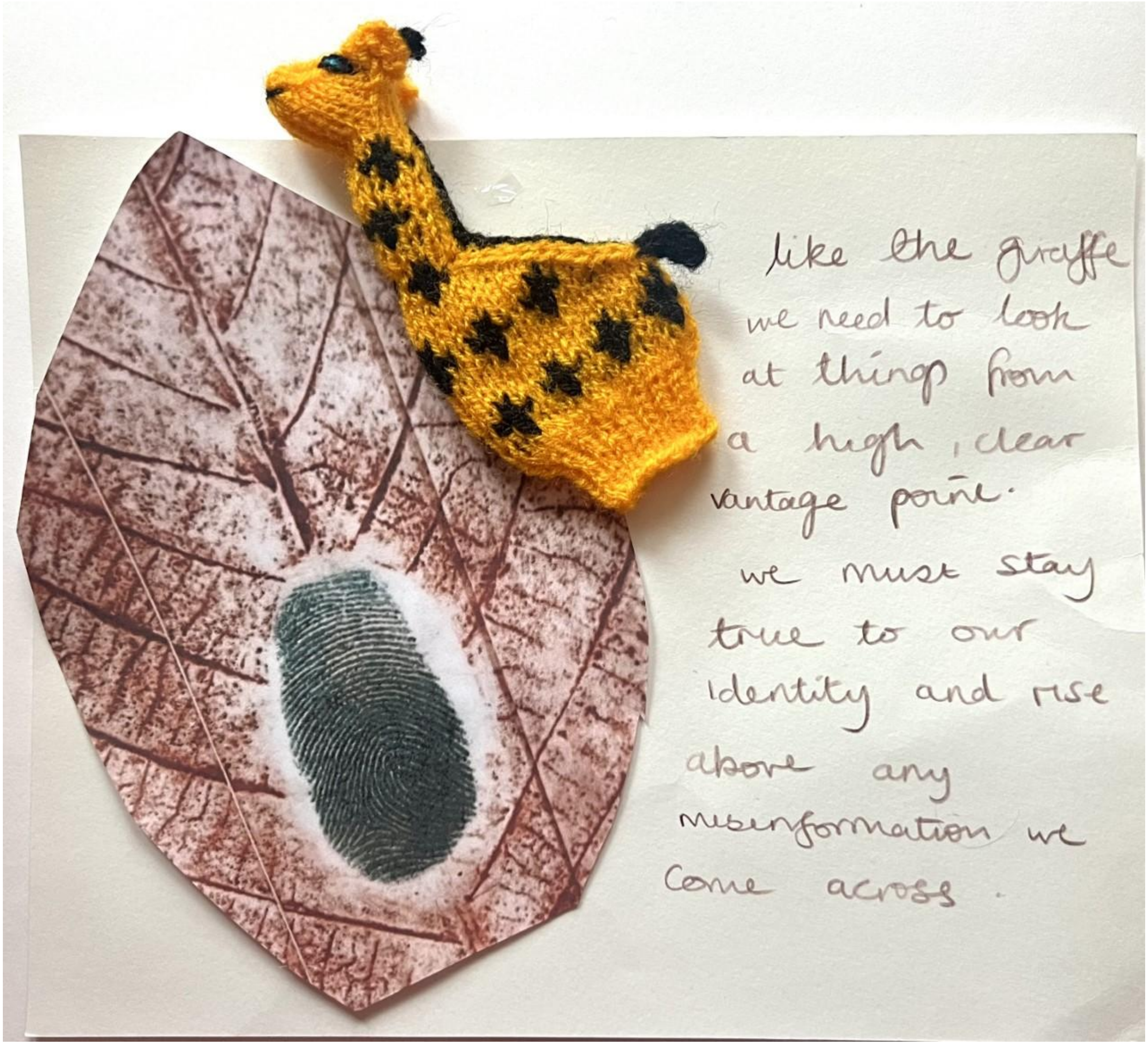
like the giraffe
we need to look
at things from
a high, clear
vantage point.
we must stay
true to our
identity and rise
above any
misinformation we
come across.

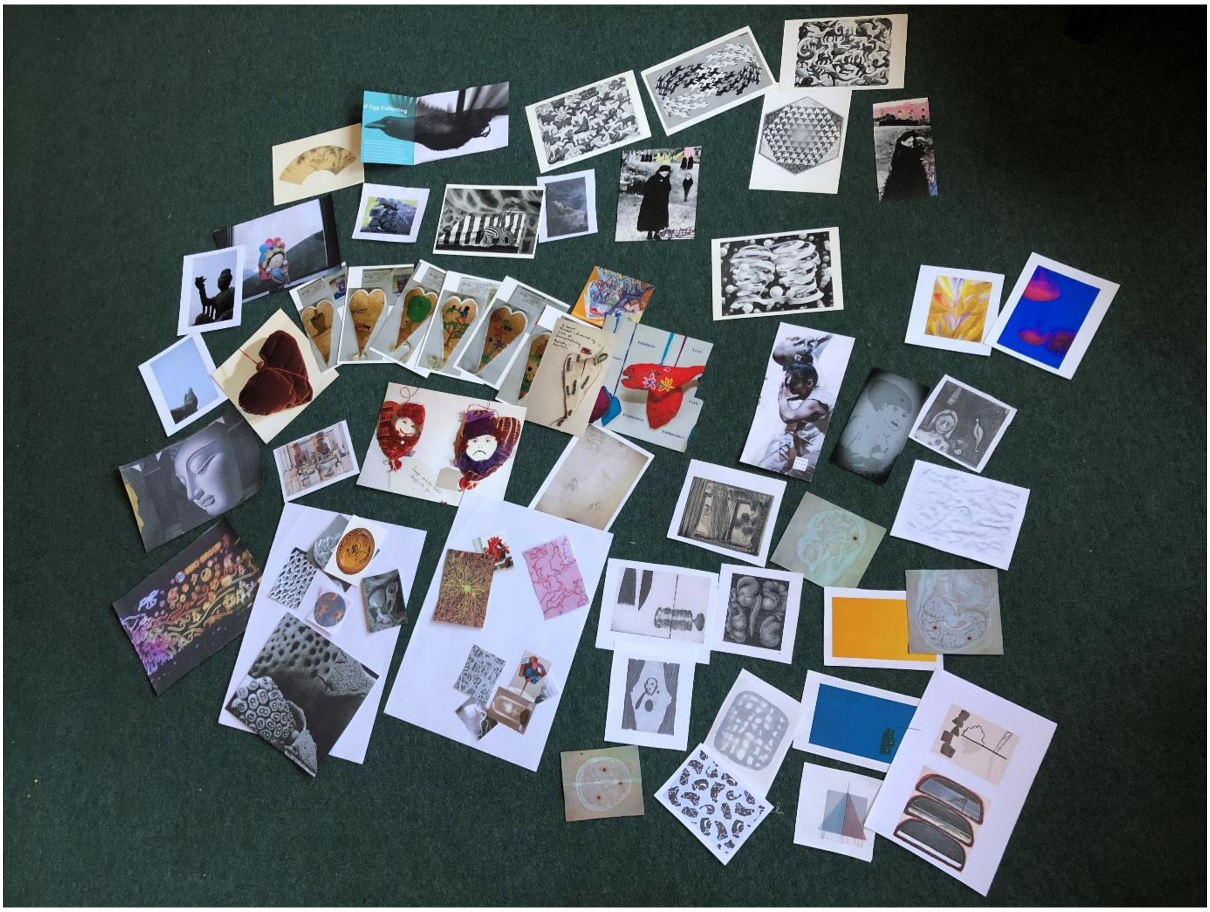

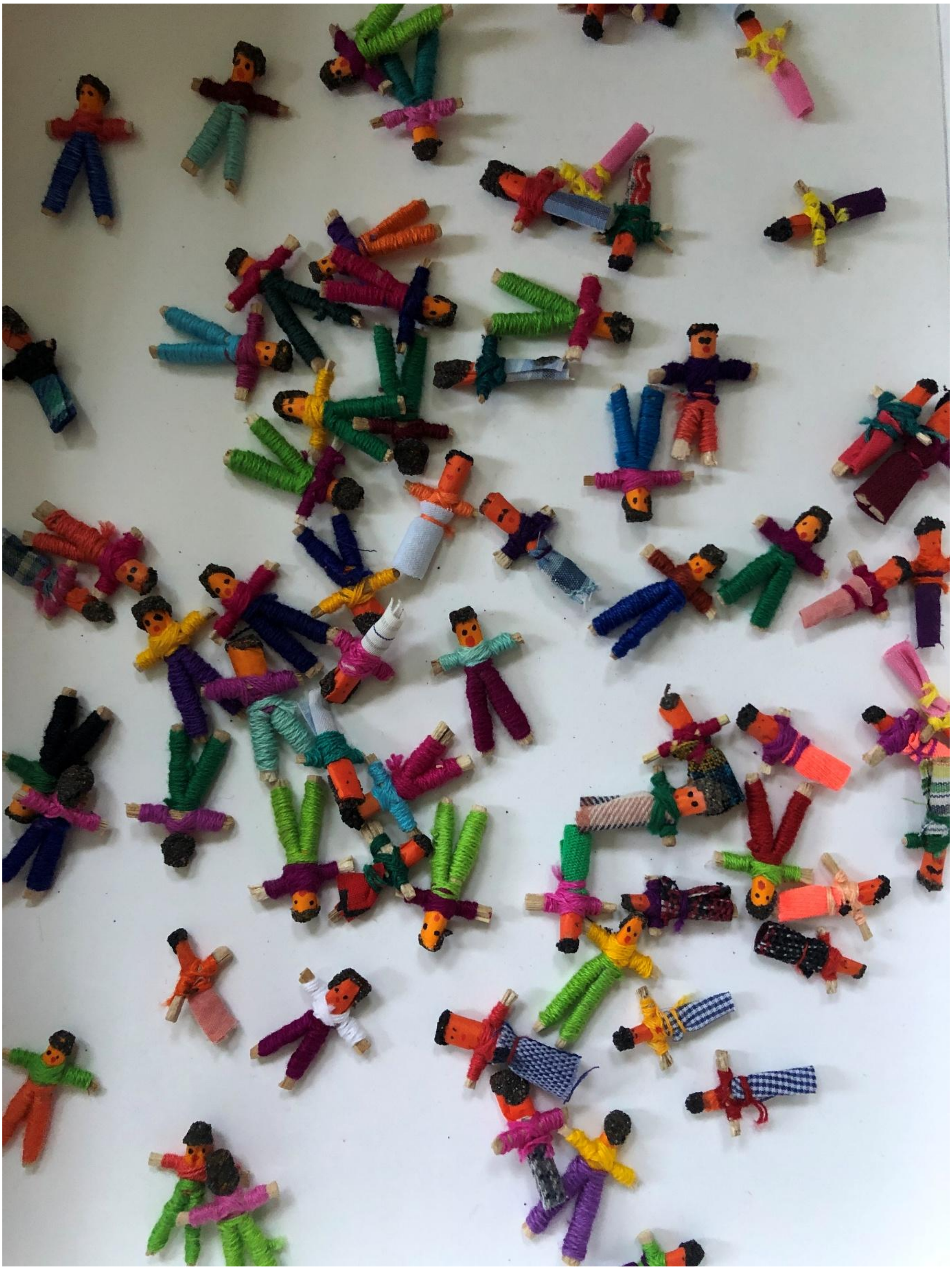

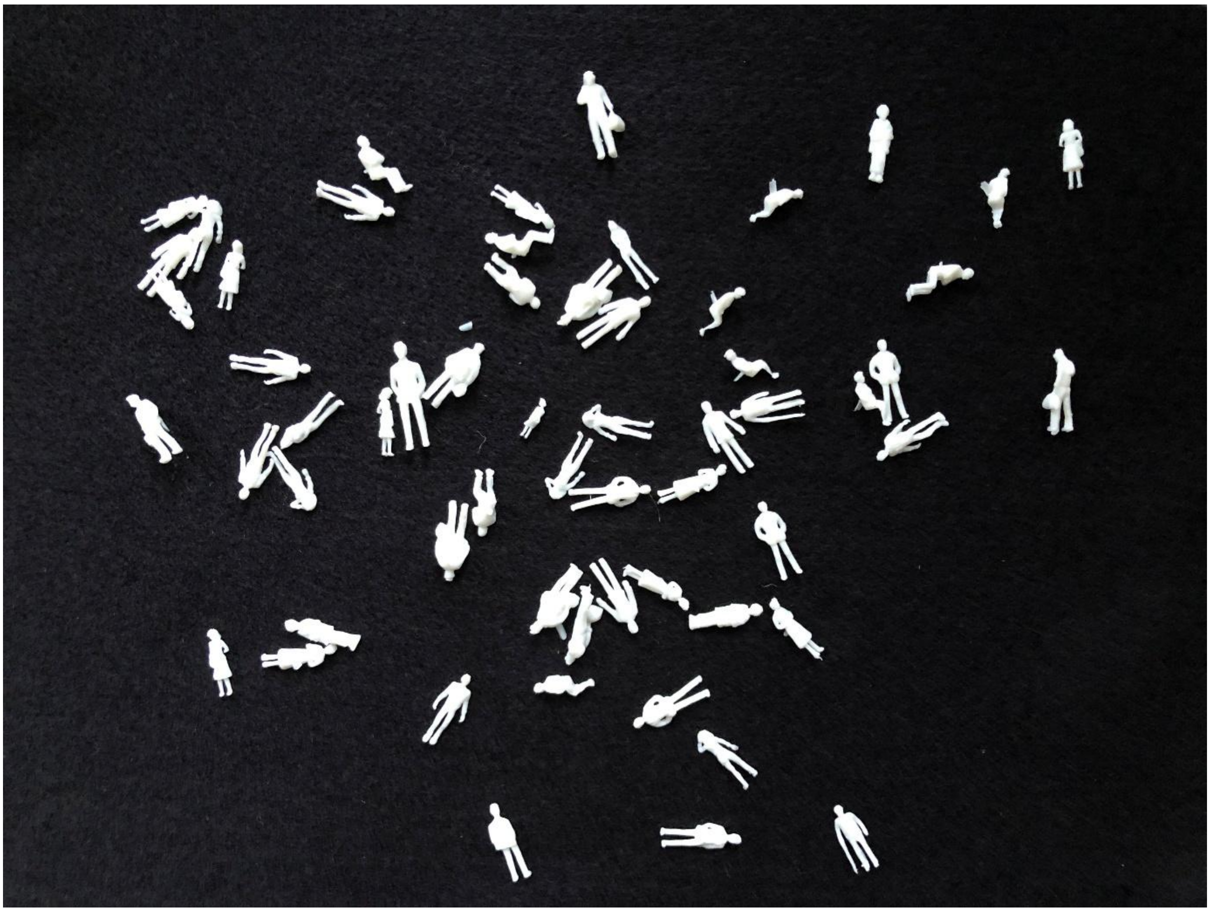

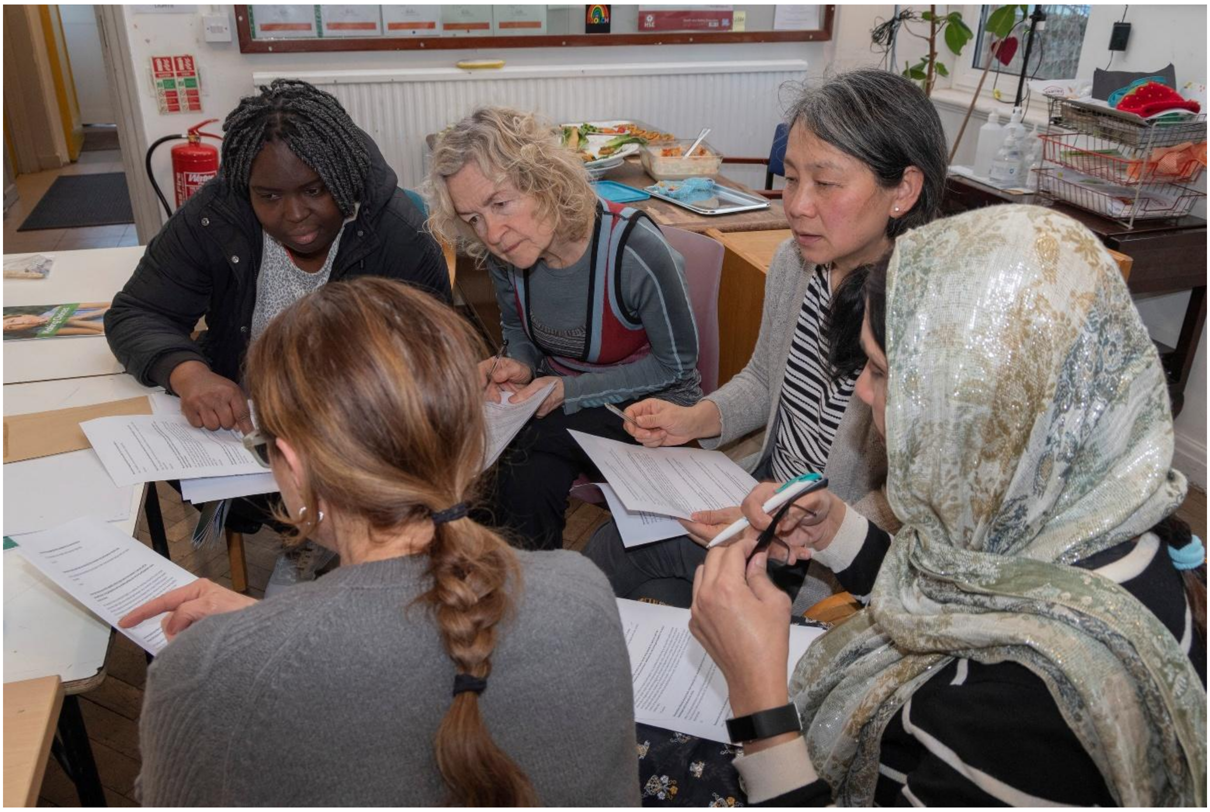

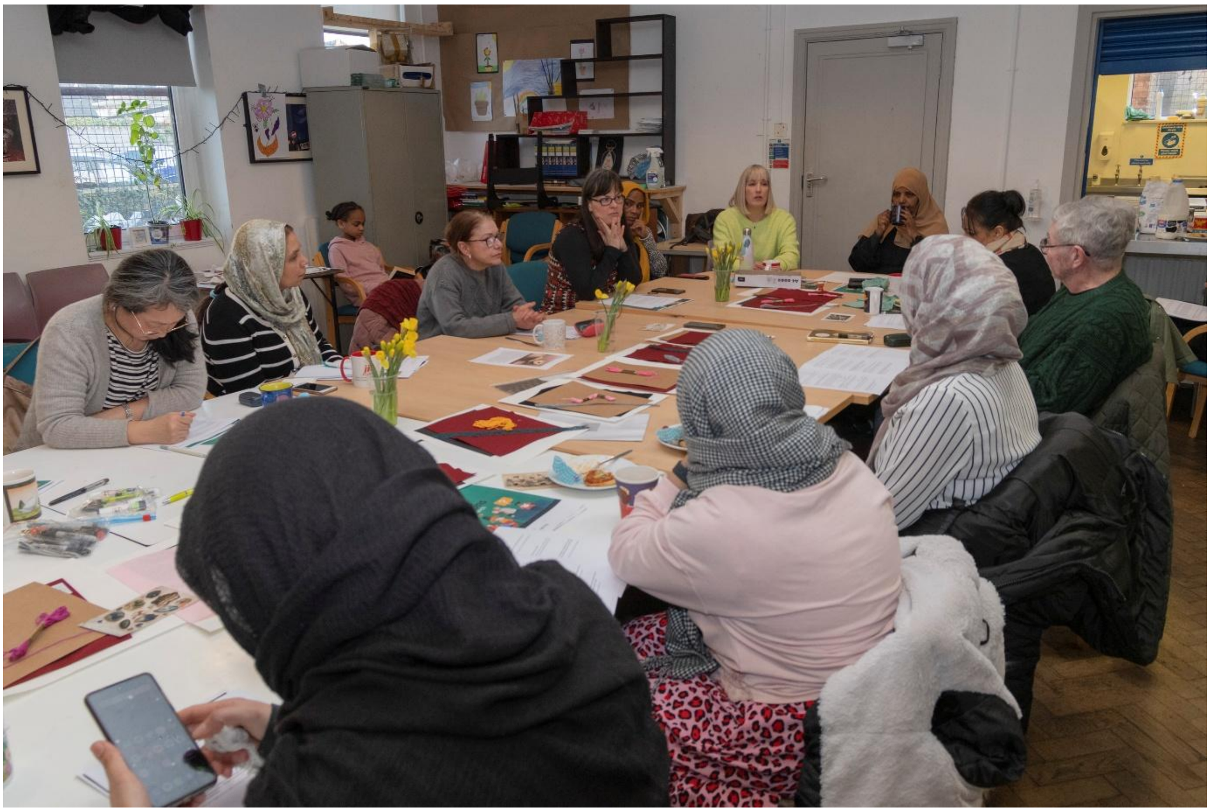

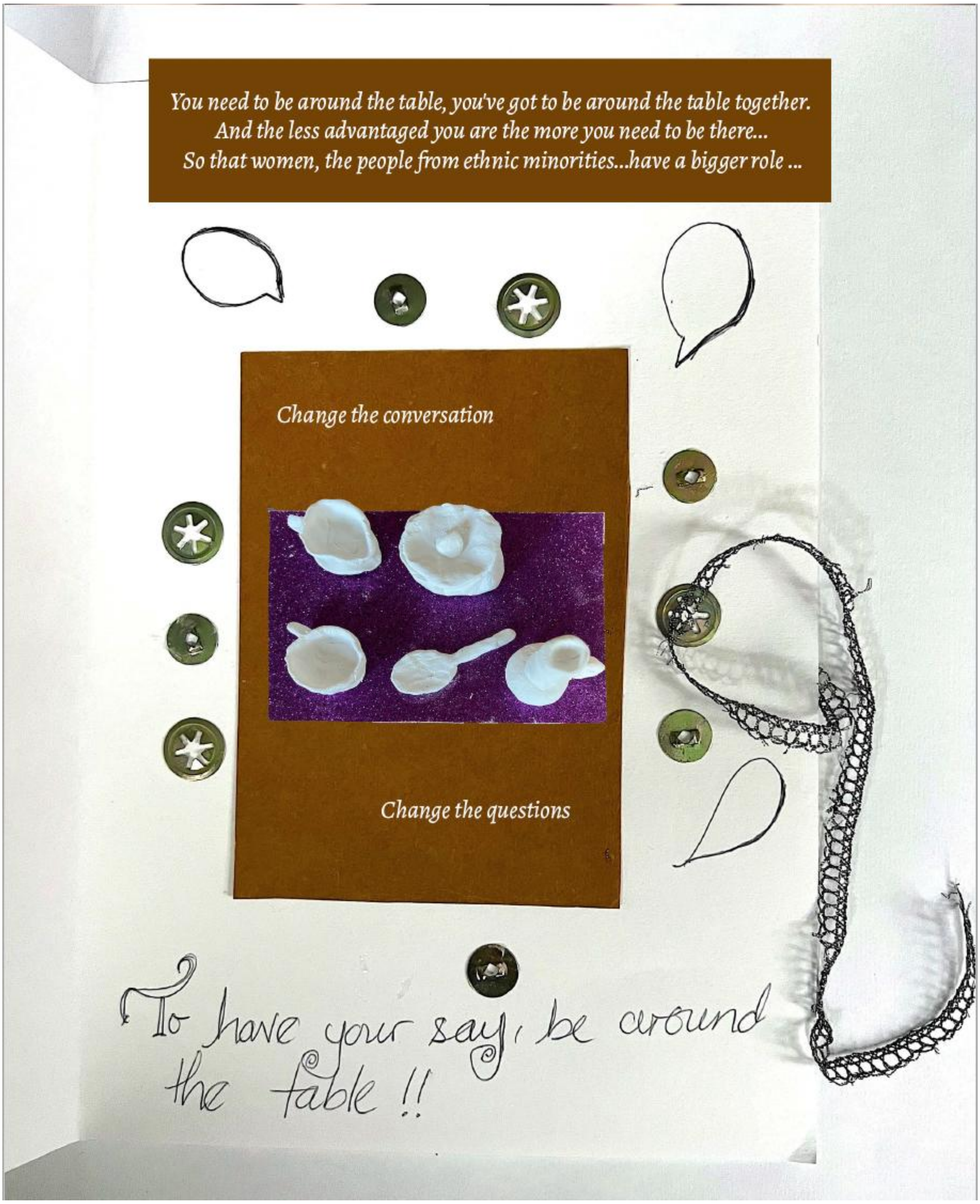
You need to be around the table, you've got to be around the table together.
And the less advantaged you are the more you need to be there...
So that women, the people from ethnic minorities...have a bigger role ...
Change the conversation
Change the questions
To have your say, be around the table !!

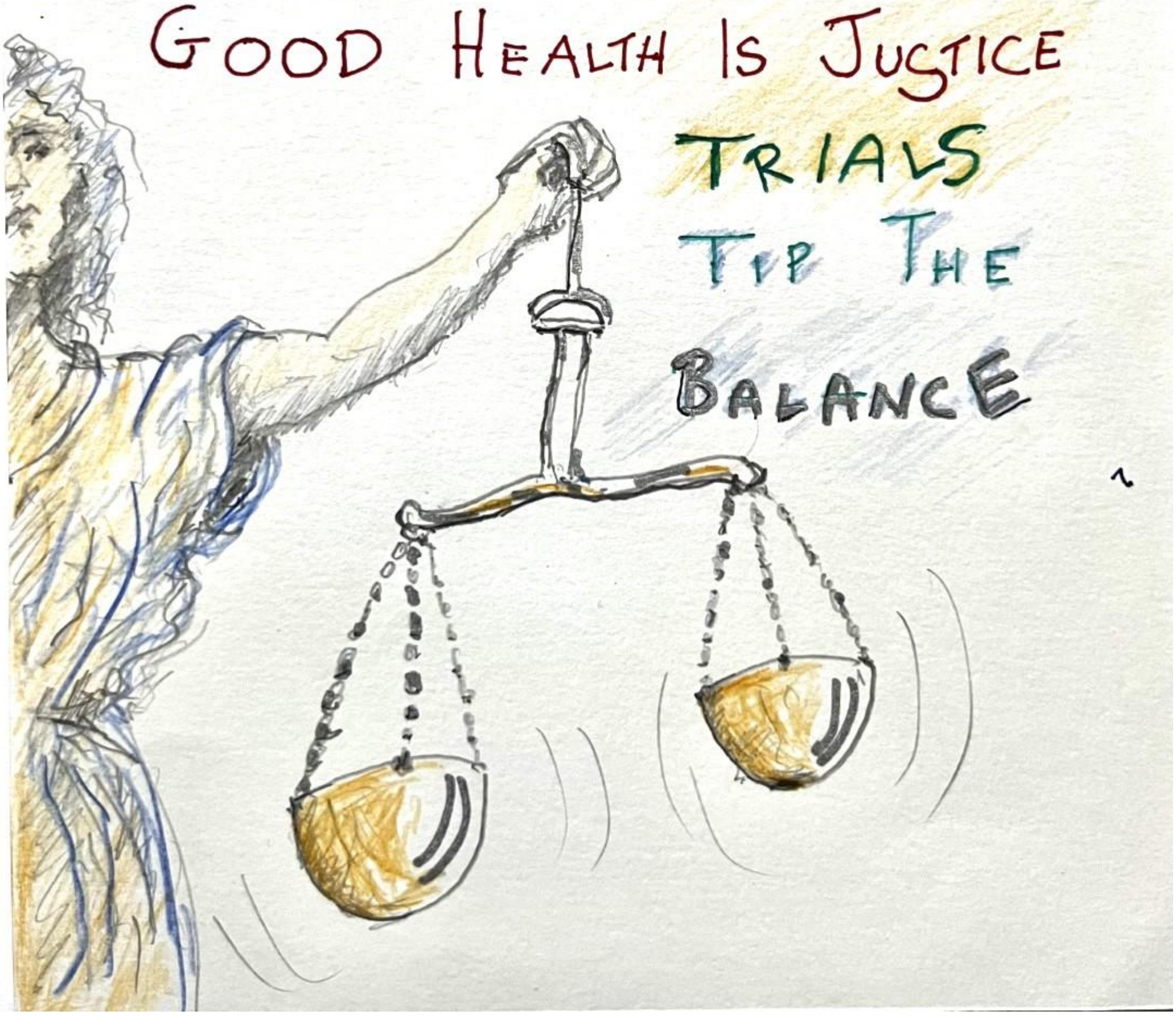
GOOD HEALTH IS JUSTICE
TRIALS
TIP THE
BALANCE

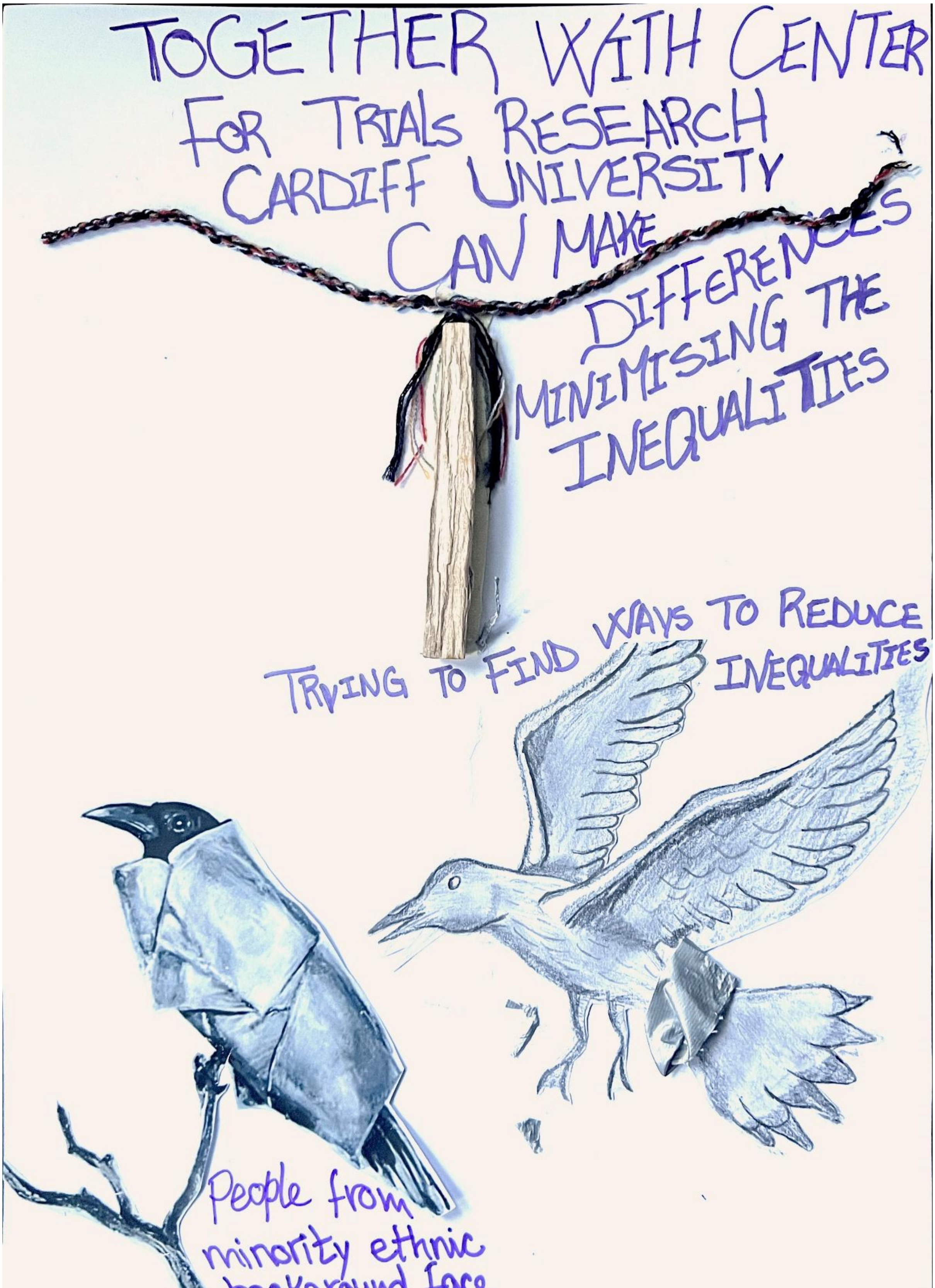
TOGETHER WITH CENTER
FOR TRIALS RESEARCH
CARDIFF UNIVERSITY
CAN MAKE
DIFFERENCES
MINIMISING THE
INEQUALITIES
TRYING TO FIND WAYS TO REDUCE
INEQUALITIES
People from
minority ethnic
background face
health inequalities.

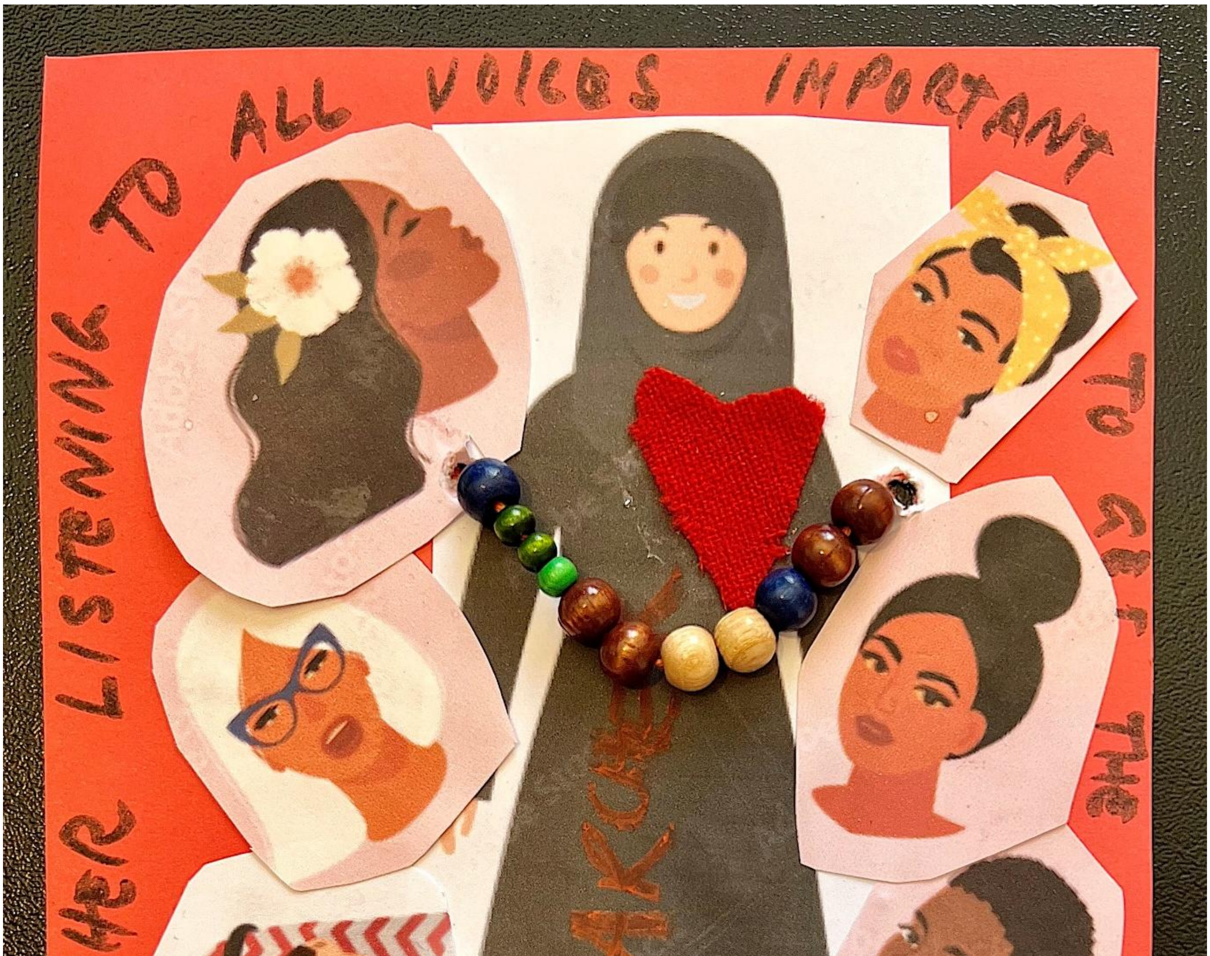
RESEARCHER LISTENING TO ALL VOICES IMPORTANT TO GET THE RIGHT ANSWER
RESEARCHER

Let’s Talk Research Workshops: Images of the creative workshop activities

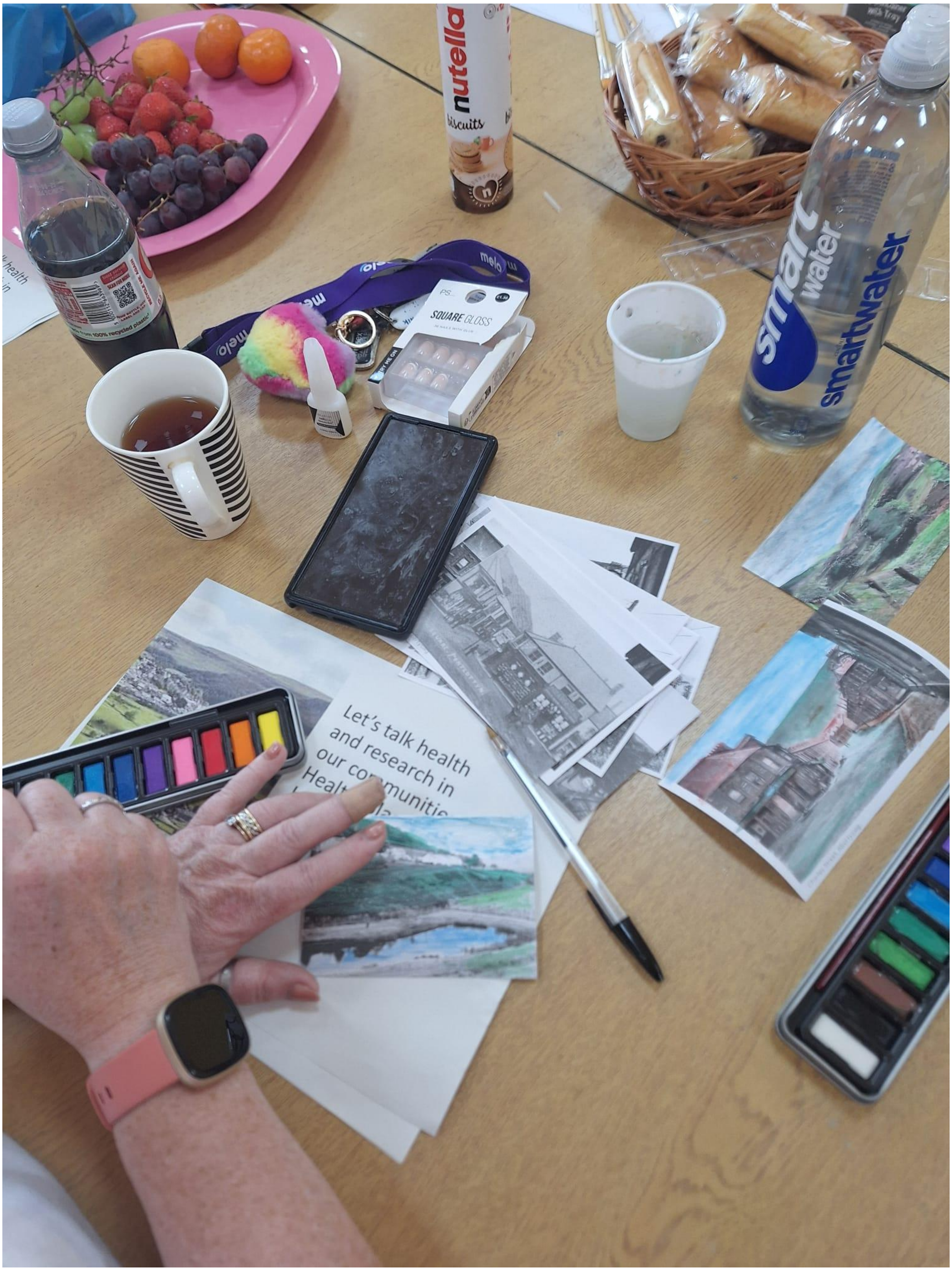

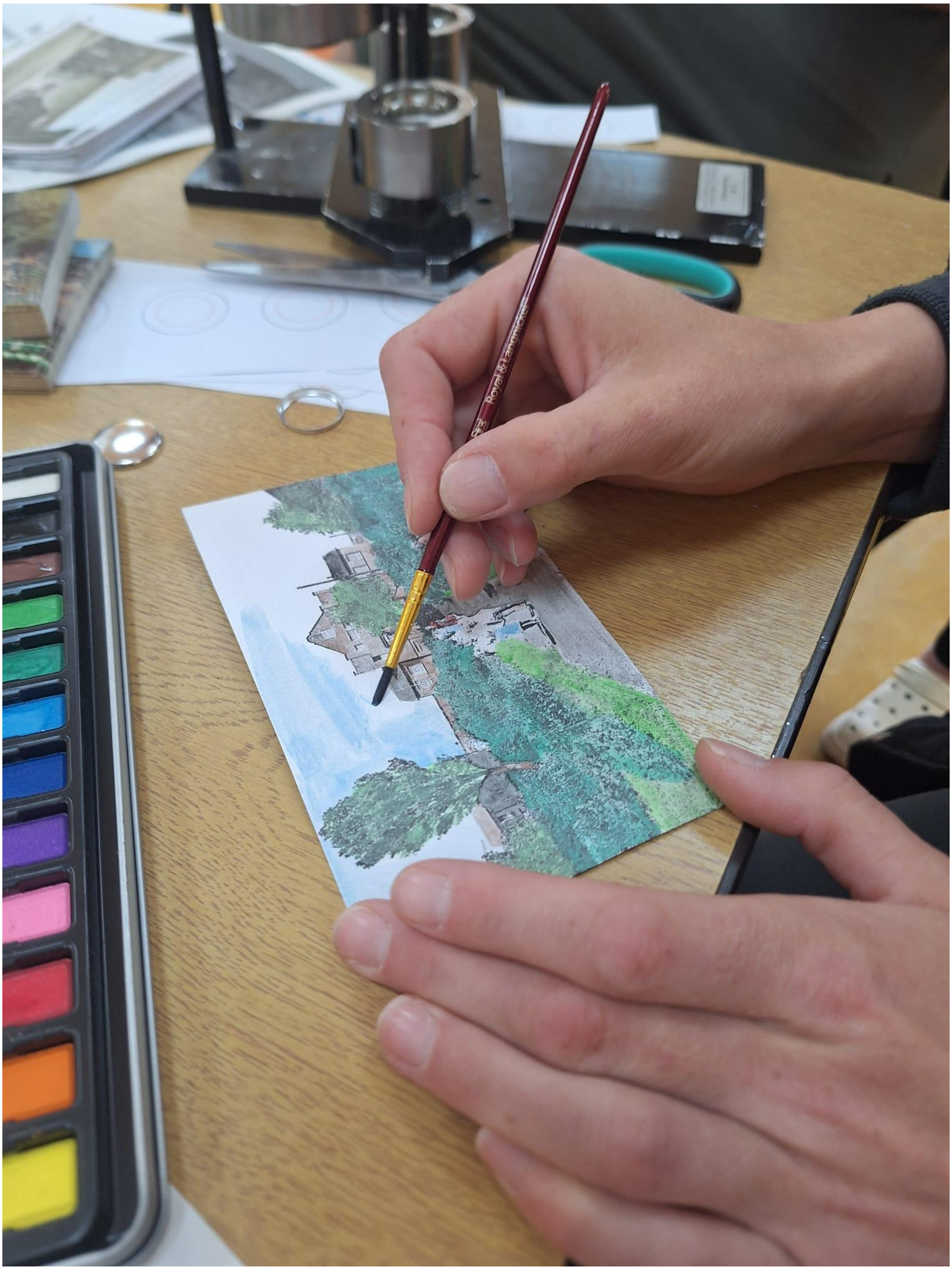
Royal & Langnickel

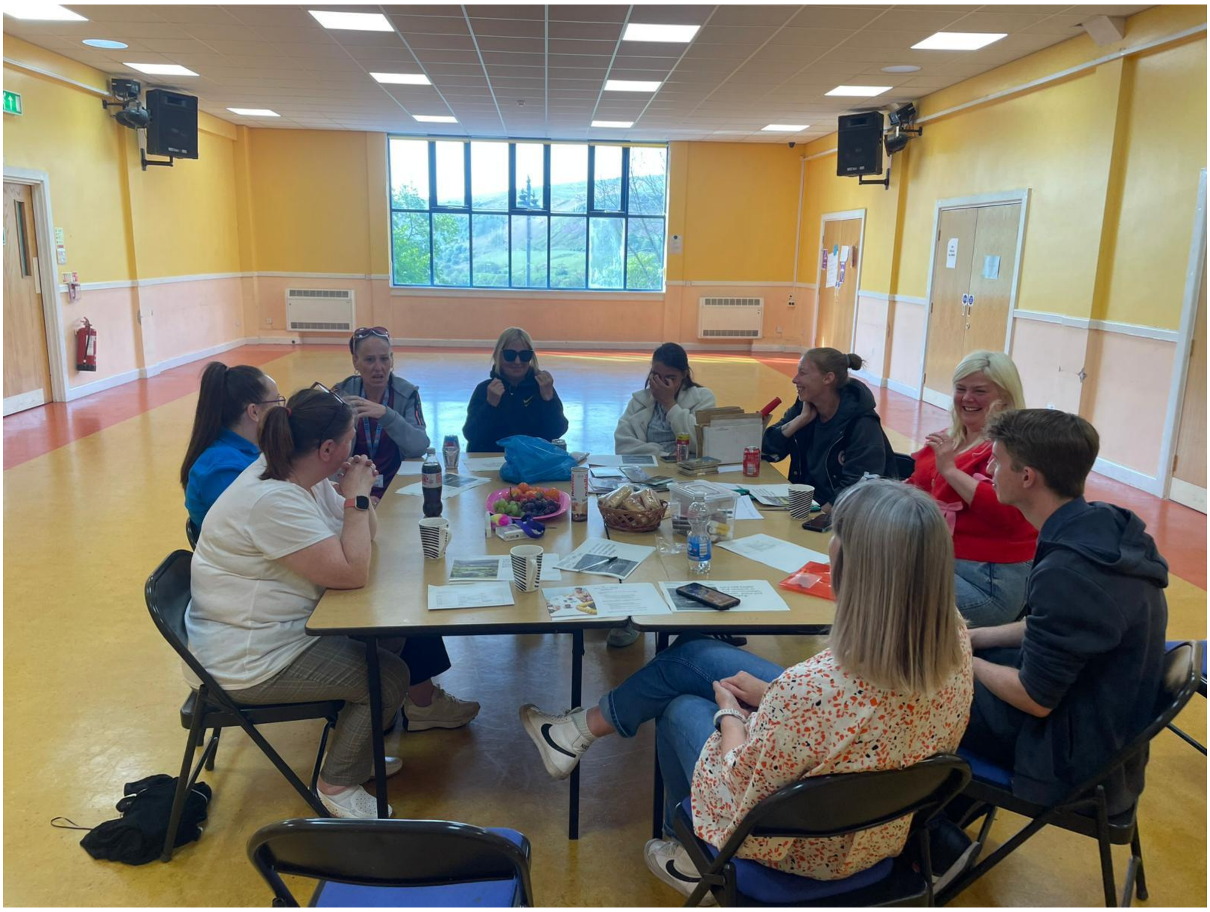

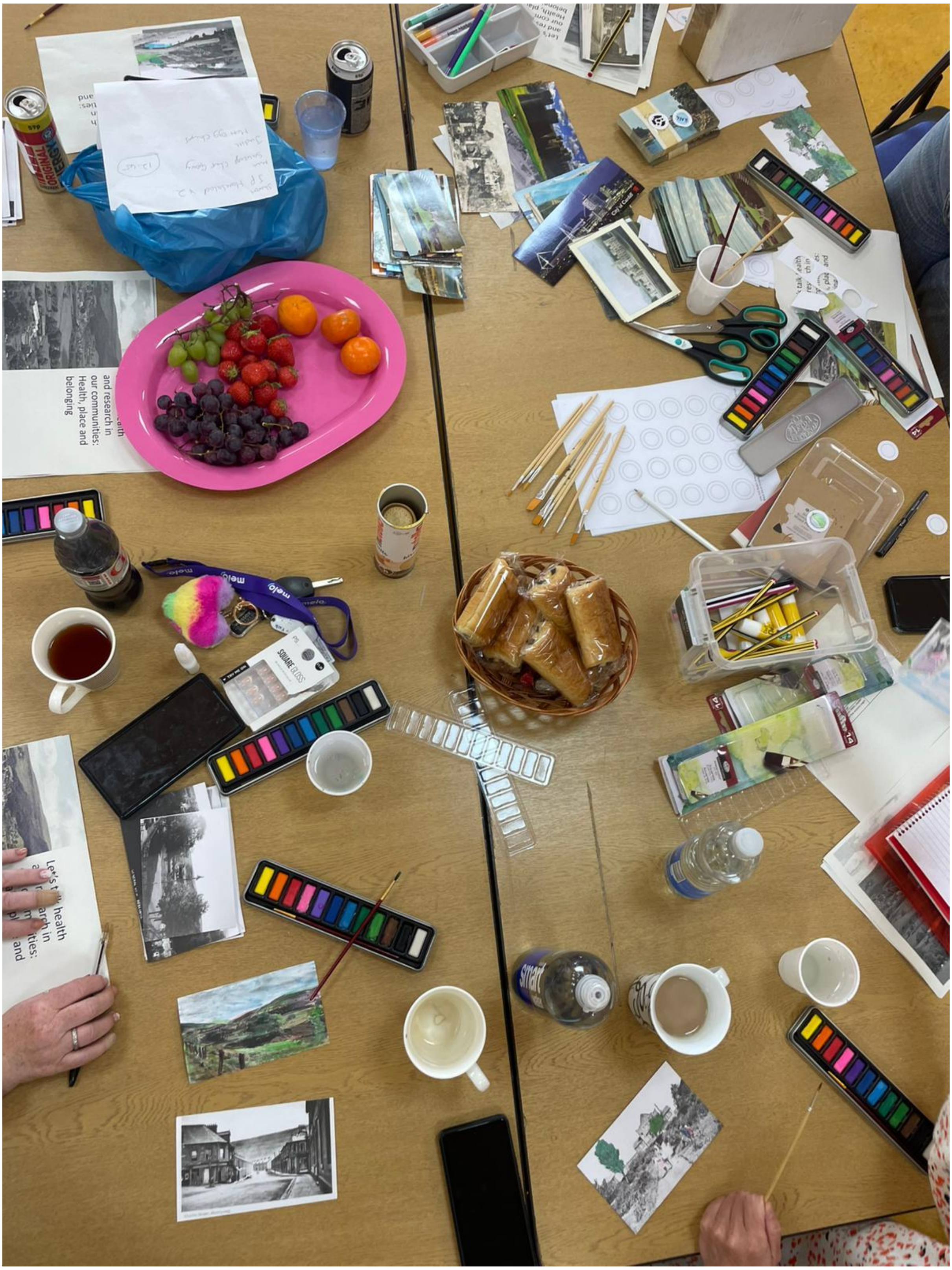
and research in
our communities:
Health, place and
belonging
SQUARE GLOSS

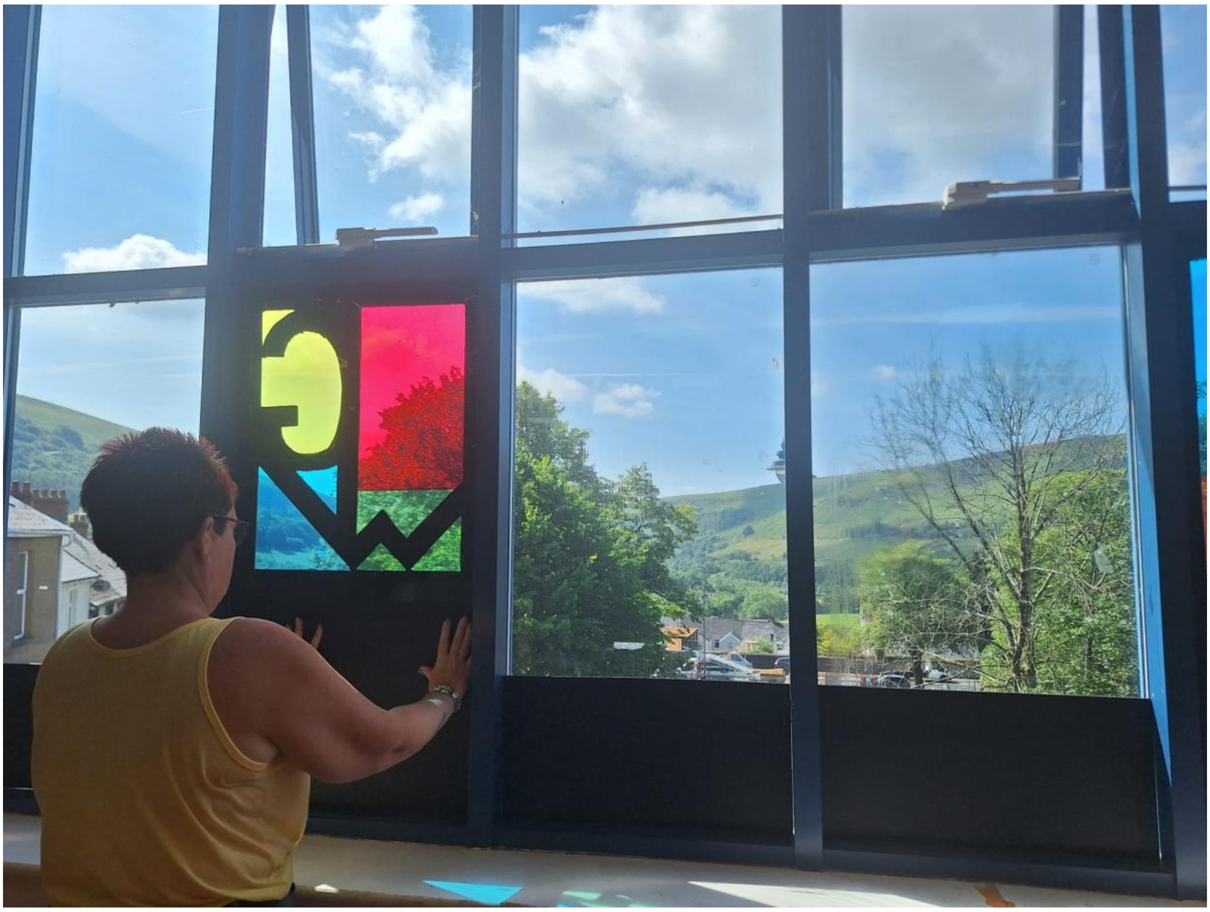

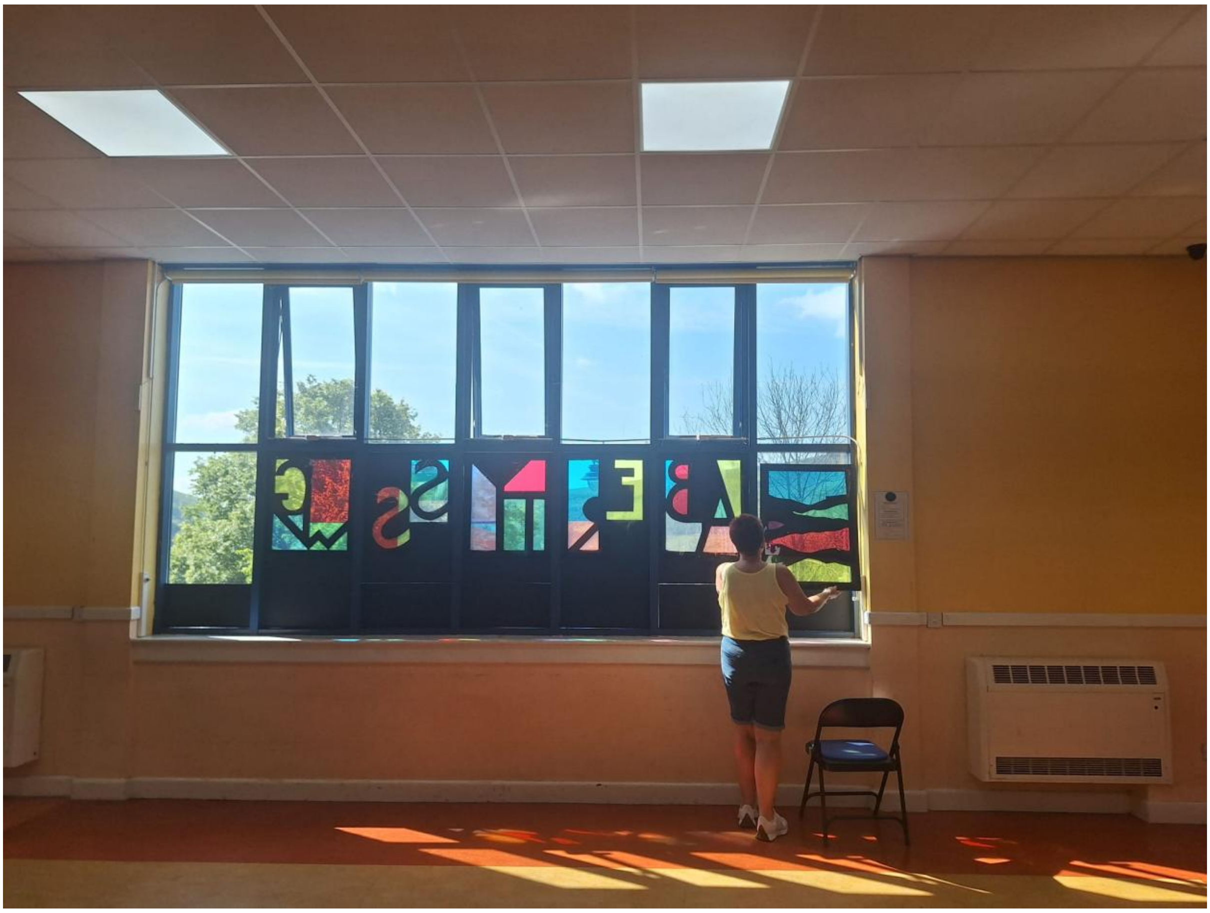

Supplement: Supplementary file 1 — Supplementary Material 1. [file 13063_2026_9820_MOESM1_ESM.pdf]
